# Improvement initiatives in the diagnostic process of heart failure: a scoping review

**DOI:** 10.3389/fcvm.2025.1681976

**Published:** 2026-01-22

**Authors:** Diego Aguiar, Rafael Gonzalez-Manzanares, Manuel Raya-Cruz, Juan Carlos Romero-Vigara, Cristina Salazar Mosteiro, Alejandro J. García Díaz, Victoria Gonzalez Pastor, Amaia Ugarte de Miguel, Eduard Ródenas-Alesina

**Affiliations:** 1 Internal Medicine Department, Heart Failure Unit, Navarra University Hospital, Pamplona, Spain; 2 Department of Cardiology, Reina Sofía University Hospital, Córdoba, Spain; 3 Instituto Maimónides de Investigación Biomédica de Córdoba (IMIBIC), Córdoba, Spain; 4 CIBER de Enfermedades Cardiovasculares, Instituto de Salud Carlos III, Madrid, Spain; 5 Internal Medicine Department, Vascular Risk Unit, Jaen University Hospital, Jaen, Spain; 6 Family Medicine, Alfajarín Primary Care Center, Health Service of Aragon, Zaragoza, Spain; 7 Instituto de Investigación Sanitaria de Aragón (IISA, Aragon Health Research Institute), Zaragoza, Spain; 8 Internal Medicine Department, Multidisciplinary Heart Failure Unit, Nuestra Señora del Prado Hospital, Talavera de la Reina, Toledo, Spain; 9 Medical Department, Heart Failure Unit, AstraZeneca, Madrid, Spain; 10 Heart Failure Unit, Cardiology Department, Vall d’Hebron University Hospital, Vall d’Hebron Institut de Recerca, Universitat Autònoma de Barcelona, Barcelona, Spain; 11 Centro de Investigación Biomédica en Red de Enfermedades Cardiovasculares, Madrid, Spain

**Keywords:** heart failure, early diagnosis, biomarkers, diagnostic imaging, artificial intelligence, care pathways

## Abstract

**Introduction:**

Heart failure (HF) poses a substantial global health burden due to its high prevalence and severe clinical outcomes. Early diagnosis is critical to optimize management and reduce the economic impact of HF. This scoping review consolidates existing knowledge on strategies to improve HF diagnosis, emphasizing the utility of biomarkers, imaging techniques, artificial intelligence (AI), and care pathways.

**Methods:**

A systematic search of PubMed/Medline and Scopus databases identified 198 relevant studies published since 2010, focusing on adult populations without a prior HF diagnosis. The inclusion criteria centered on initiatives aimed at enhancing diagnostic processes.

**Results:**

Results indicate that biomarkers, particularly natriuretic peptides such as N-terminal prohormone of BNP (NT-proBNP), are central to early HF detection, showing high sensitivity. Emerging biomarkers, like microRNAs, offer potential for improved diagnostic accuracy. Imaging techniques, including echocardiography and lung ultrasound, remain primary tools for assessing cardiac function, while AI applications in imaging and electronic health records represent a rapidly evolving field. These tools show promising potential for early identification of HF patients, although most require further validation and standardization before routine clinical implementation. Care pathways emphasizing high-resolution consultations and integrated diagnostic tools enable prompt HF diagnosis, crucial for initiating early treatments.

**Discussion:**

By implementing these diagnostic strategies, particularly in high-risk populations such as those with comorbid conditions, there is potential to significantly advance patient outcomes and healthcare resource management. Nevertheless, it is essential to translate these advances and discoveries into clinical practice, considering healthcare context and socioeconomic limitations, and promoting international consensus to ensure their global adoption. In conclusion, ongoing research and refinement of these diagnostic tools are imperative to effectively address the growing challenge of HF.

## 1 Introduction

Heart failure (HF) constitutes a major global health challenge, characterized not only by its high prevalence but also by the severe clinical and economic consequences it entails. It is estimated to affect 2%–3% of the general adult population, reaching up to 10% in individuals over 70 years of age, with projections indicating continued growth (1). This increase is attributed to the aging of the population, because of longer life expectancy, the growing prevalence of HF risk factors such as diabetes and hypertension, and technological and therapeutic advances that enhance the prevention of cardiovascular events through early diagnosis and treatment (2). The increase in the prevalence of HF has been consistently reported across different countries (3), further highlighting concerns about the expansion of this pathology in the coming years.

Although the emergence of new therapies has helped alleviate some of the burden of HF, mortality and hospitalization rates remain alarmingly high. Indeed, 20% of patients die within the first year following diagnosis, and nearly 50% within the first five years, underscoring its severity (4). HF-related hospitalizations account for more than 25% of total hospital admissions in Spain, making it the leading cause of hospitalization in individuals over 65 years of age (4). This situation underscores the urgent need for earlier and more effective therapeutic strategies (4).

In addition to mortality, reducing HF hospitalizations is a crucial target in the treatment of HF, as they significantly contribute to the clinical deterioration of patients. In this regard, the prompt initiation of treatment after diagnosis is paramount for achieving the best improvement of the disease prognosis (5). National observational studies have shown that a considerable proportion of patients do not receive optimal treatment, highlighting the existing gap between current clinical practice and therapeutic guidelines (5).

Besides the clinical implications, the relevance of an early diagnosis also lies in the economic consequences of HF, with hospitalizations representing a major component of HF-related costs (6, 7). As has been demonstrated, early initiation of guideline-recommended treatments improves clinical outcomes, resulting in a significant reduction in hospitalizations and related costs in the long term. This reinforces the need to promote early intervention strategies to address both the clinical and economic impact of HF (8–10).

This publication aims to consolidate knowledge about strategies that enable screening and, consequently, early diagnosis of HF, allowing for the timely initiation of treatment. To achieve this, a scoping review of the current literature has been conducted, which we have opted to divide into sections corresponding to the main diagnostic and screening approaches currently explored for identifying HF patients: Biomarkers, Imaging Techniques, Artificial Intelligence (AI), and Care Pathways.

## 2 Methods

A scoping review was conducted to identify, assess, and synthesize initiatives aimed at improving the diagnostic process for HF globally since 2010. The review initially focused on six predefined areas: (1) biomarkers; (2) imaging techniques; (3) AI; (4) devices; (5) high risk of HF; (6) clinical suspicion of HF.

### 2.1 Search strategy and eligibility criteria

Searches were performed in PubMed/Medline and Scopus databases, with the last search conducted in July 2024. The search terms and complete strategies for each of the six predefined areas are detailed in Supplementary Tables S1, S2. Grey literature and preprints were not included. Peer-reviewed articles published in English or Spanish describing initiatives aimed at improving the diagnostic process for HF in adult populations were considered eligible. Studies were excluded if they reported preclinical and animal models, studies including pediatric populations, studies published in languages other than English or Spanish, studies including comorbidities unrelated to HF (e.g., amyloidosis, aortic stenosis, cancer, etc.) or innovations in other phases of HF care (hospital discharge, transitional care, long-term follow-up, treatment etc.).

### 2.2 Study selection

Two reviewers were involved in the screening of the references: one made the decisions, and the other reviewed the selection. The whole process was supported through the Covidence platform (https://www.covidence.org). References identified from the searches in Scopus and PubMed were merged in Covidence and duplicates were eliminated. Subsequently, references were screened by title and abstract, and those that met eligibility criteria were maintained for full text review. The same inclusion and exclusion criteria were applied during the full-text review to ensure consistency in study selection. If authors came across relevant references that had not previously been identified by the systematic search, they included them as results of a manual search, provided they met the selection criteria.

The whole process was documented as recommended by the Preferred Reporting Items for Systematic Reviews and Meta-Analyses extension for Scoping Reviews (PRISMA-ScR) (11).

### 2.3 Data extraction

Data from the included articles were reviewed qualitatively. The articles were organized by thematic areas in an Excel spreadsheet, with each author responsible for reviewing the articles corresponding to one specific area of the review. For each article, relevant details were documented, including study design, type of HF classification based on left ventricular ejection fraction (LVEF), healthcare setting (e.g., primary care, emergency department), and the diagnostic initiative or strategy proposed. Extracted data from the included studies were compiled in a standardized table summarizing their main characteristics (Supplementary Table S3).

### 2.4 Data synthesis

Data synthesis was narrative. Although the review initially considered six areas, the evidence was ultimately grouped and synthesized into four main sections: (1) biomarkers, (2) imaging techniques, (3) AI, and (4) care pathways. Within each section, findings were summarized according to their relevance and potential impact on improving HF diagnosis, considering both the type of strategy and the healthcare setting in which it was intended to be used.

## 3 Results

**Figure 1:**
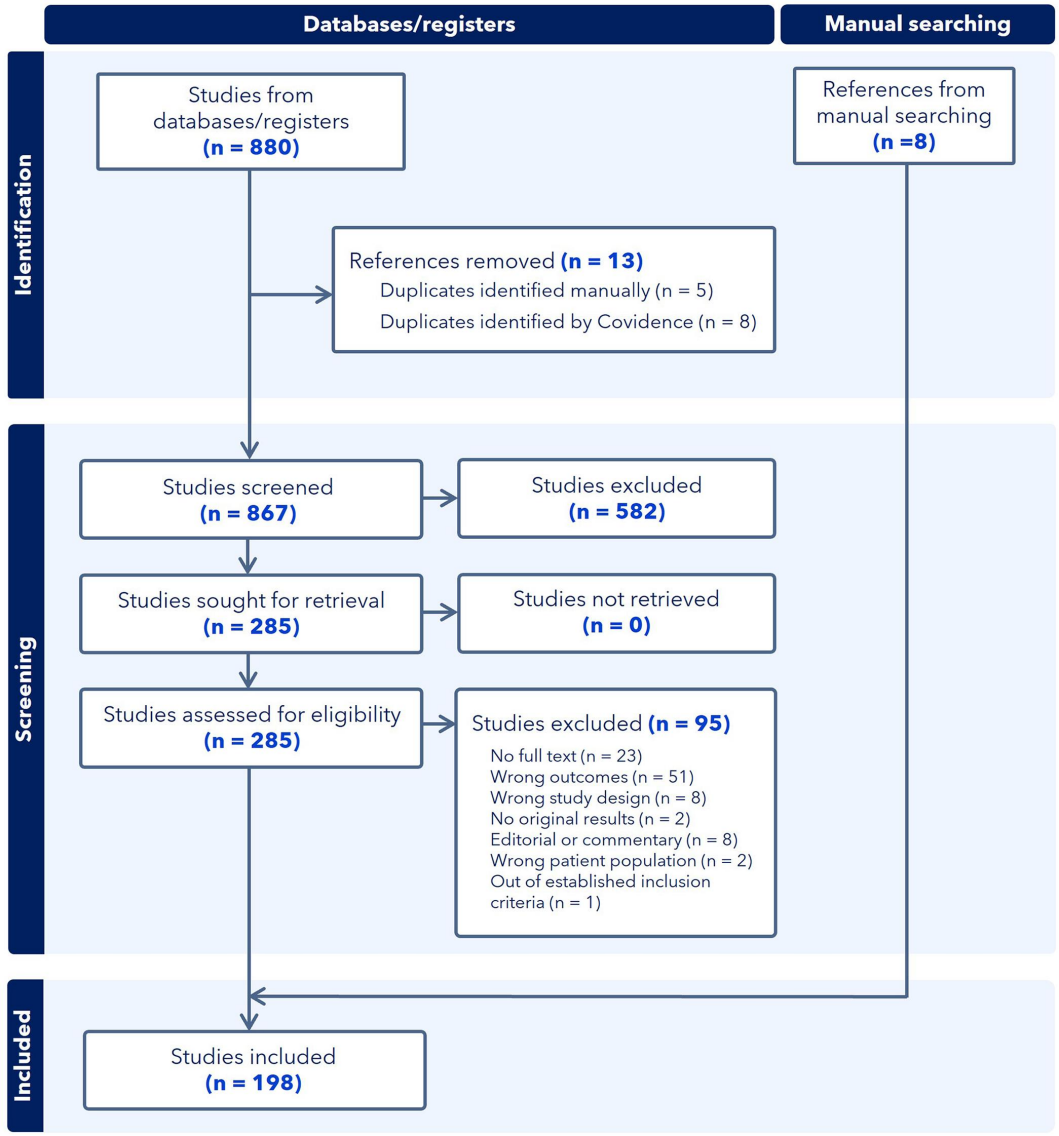
PRISMA flow diagram of literature and study selection.

We identified 880 studies, of which 198 met the inclusion criteria for this scoping review. A summary of the characteristics of the included studies is provided in Supplementary Table S3, and the study selection process is illustrated in the PRISMA flow diagram (Figure 1).

### 3.1 Biomarkers: natriuretic peptides in HF diagnosis

From a total number of 198 studies, 96 (48.5%) focused on biomarkers. Of these, 29 key studies specifically addressed the use of natriuretic peptides as screening tool for HF, of which 12 (41.4%) evaluated the detection of brain natriuretic peptide (BNP) and N-terminal prohormone of BNP (NT-proBNP), 3 (10.3%) also included atrial natriuretic peptide (ANP), and 12 (41.4%) assessed only NT-proBNP. Approximately one third of the studies (34.5%) included patients admitted to an emergency department with suspected acute HF. A minor fraction (13.8%) evaluated their utility within a diagnostic-therapeutic process or protocol. Most of studies are observational, with prospective (55.2%), retrospective (13.8%), and cross-sectional designs (3.5%). Rest of the studies comprises meta-analyses, systematic reviews and literature reviews. In 11 studies (37.9%), the relation between natriuretic peptides and ventricular ejection fraction was investigated, with two specifically focusing on patients with preserved ejection fraction (HFpEF).

Natriuretic peptides are a group of neurohormones classified into three subtypes: A, B, and C. ANP and BNP are produced in the myocardium, whereas type C is synthesized in mesothelial cells. However, only ANP, BNP, and more specifically NT-proBNP are measurable in the blood (12). NT-proBNP is the most commonly used in clinical laboratories due to its longer biological half-life (up to 72 h) and the rapid availability of results (15 min) (12). Although its utility for HF diagnosis has shown promising results in other biological fluids, such as urine (13), pleural fluid (14), and saliva (15), this review focuses on the benefits of serum NT-proBNP in the differential diagnosis of patients with dyspnea.

NT-proBNP has shown high sensitivity for HF diagnosis in patients presenting with new-onset dyspnea, both in outpatient settings in primary care (16–21) as in emergency departments (22–24). Furthermore, it is useful in identifying new-onset HF in patients with known atrial fibrillation (AF) (25).

In the emergency department, plasma levels of natriuretic peptides should be measured in all patients with acute dyspnea and suspected acute HF to help in the differential diagnosis from other potential causes (26, 27). Guideline recommendations define cut-offs of 100 pg/mL for BNP and 300 pg/mL for NT-proBNP for the diagnosis of acute HF (9, 27). However, their diagnostic use performs better for ruling out HF than for confirming it, as elevated natriuretic peptide values are associated with a wide range of cardiac and non-cardiac conditions (9, 27). Specificity increases when combined with other tests, such as electrocardiography, chest x-ray, transthoracic echocardiogram, or pulmonary computed tomography (CT) (28, 29). Age-adjusted NT-proBNP cut-offs have been proposed and appear promising in patients with acute dyspnea: ≥450 pg/mL for those under 50 years, ≥900 pg/mL for those between 50 and 75 years, and ≥1,800 pg/mL for those over 75 years (26, 30), although these have not yet been formally adopted by international guidelines.

In the non-acute setting, plasma levels of natriuretic peptides can be used as an initial diagnostic test, identifying those patients who require further cardiac assessment (27). Current guidelines define upper limits of normal as 35 pg/mL for BNP and 125 pg/mL for NT-proBNP, with negative predictive values ranging from 0.94 to 0.98 for concentrations below these thresholds (9, 27). However, some patients with NT-proBNP <125 pg/mL could have HF, but it should be noted that these are exceptional cases (31). In contrast, there is no well-defined or universally accepted rule-in thresholds for natriuretic peptides in the outpatient setting (9, 27).

In addition, several limitations may affect the interpretation of natriuretic peptide levels (25–29). NT-proBNP levels typically fluctuate throughout the day (32), are reduced in obese individuals (33–35) and are elevated in patients with chronic kidney disease (CKD) (36), pulmonary hypertension of non-cardiac origin (37) or other structural cardiac conditions such as valvular diseases (37) or AF (38). Since NT-proBNP levels can differ in these conditions, different cut-off values may need to be considered. For example, in patients with AF, the NT-proBNP cut-off should be increased by 50% when the ventricular rate is ≤90 bpm at the time of the blood draw or by 100% when the ventricular rate is >90 bpm (30). Furthermore, in this population, a cut-off of >365 pg/mL has been recommended for the diagnosis of HFpEF in the non-acute setting (9). Table 1 summarizes natriuretic peptide cut-offs by setting (emergency department vs. non-acute/outpatient) and highlights context-specific considerations such as age, AF, CKD, and obesity.

**Table 1:** Natriuretic peptide cut-offs by setting and evidence base.

Context	Cut-off	Evidence type	Comments
Emergency department (ED)	AHF rule-out: NT-proBNP <300 pg/mL; BNP <100 pg/mL	Guideline	ESC 2021 HF; Recommended to rule out acute HF (9)
	AHF rule-in: Age-adjusted cut-off: 450 pg/mL (<50 years); 900 pg/mL (50–75 years); 1,800 pg/mL (>75 years)	Study-derived/ HFA–ESC clinical consensus (not guideline)	Proposed age-adjusted cut-offs; supports rule-in but not universally recommended by guidelines (30)
	Specific conditions (AF, CKD, obesity): Do not necessitate additional adjustments	Study-derived/ HFA–ESC clinical consensus (not guideline)	No guideline-endorsed adjusted NP cut-offs in ED (30)
Non-acute/outpatient setting	HF triage: NT-proBNP 125 pg/mL; BNP 35 pg/mL	Guideline	ESC 2021 HF; Below cut-off: HF unlikely; above: justify further evaluation (9)
	Age adjustment: ≥125 pg/mL (<50 years); ≥250 pg/mL (50–75 years); ≥500 pg/mL (>75 years)	Study-derived/ HFA–ESC clinical consensus (not guideline)	Not universally adopted by official guidelines; needs further validation (30)
	Obesity adjustment: Proposed reductions vs. standard: −25% (BMI 30–34.9); −30% (35–39.9); −40% (≥40)	Study-derived/ HFA–ESC clinical consensus (not guideline)	NP reduced in obesity; not universally adopted by official guidelines; needs further validation (30)
	AF adjustment: Proposed increases vs. standard: +50% (ventricular rate ≤90 bpm); +100% (ventricular rate >90 bpm)	Study-derived/ HFA–ESC clinical consensus (not guideline)	AF elevates NP; not universally adopted by official guidelines; needs further validation (30)
	CKD adjustment: Proposed increases vs. standard: + 35% (eGFR <30); +25% (eGFR 30–45); +15% (eGFR 45–60)	Study-derived/ HFA–ESC clinical consensus (not guideline)	CKD elevates NP; interpret cautiously; not universally adopted by official guidelines; needs further validation (30)

AF, atrial fibrillation; BMI, body mass index; BNP, B-type natriuretic peptide; CKD, chronic kidney disease; ED, emergency department; eGFR, estimated glomerular filtration rate; ESC, European Society of Cardiology; HF, heart failure; HFA, Heart Failure Association; NT-proBNP, N-terminal pro–B-type natriuretic peptide; NP, natriuretic peptide.

Moreover, sex-related differences in natriuretic peptide levels have been described, with women generally exhibiting slightly higher BNP and NT-proBNP concentrations at comparable clinical status. However, current guidelines do not recommend sex-specific cut-offs due to insufficient evidence regarding their impact on diagnosis and prognosis (30, 39).

### 3.2 Other biomarkers: emerging biomarkers for HF diagnosis

Among all included articles, 17 (8.6%) provided information on other biomarkers (other than natriuretic peptides), of which 9 (52.9%) described interesting strategies to improve HF diagnosis. Specifically, 3 out of the 9 (33.3%) examined suspected acute HF in the emergency department, while 4 (44.4%) focused on suspected chronic HF. Additionally, 2 studies (22.2%) assessed at-risk populations, such as obese patients or those with CKD. Regarding the type of HF classification by LVEF, 5 out of 9 (55.6%) included both patients with HFpEF and HF with reduced ejection fraction (HFrEF), 1 (11.1%) included only patients with HFrEF, 1 (11.1%) included only patients with HFpEF, and 2 (22.2%) did not specify the type of HF. Most of the studies are observational, mainly prospective (66.7%).

As mentioned in the previous section, BNP and NT-proBNP stand out for their diagnostic utility, being the most widely used biomarkers in HF due to their high sensitivity and negative predictive value. However, their efficacy can be influenced by different factors (25–29). Therefore, there is a need for more specific diagnostic biomarkers, among which small non-coding RNA molecules with an important role in regulating gene expression (microRNAs) have recently emerged as a promising option.

MicroRNAs play an important role in cardiac differentiation, proliferation, and maturation, which has led to their potential use in various cardiovascular diseases, including HF (40). They are released into the circulation and are highly stable in plasma, making them detectable via polymerase chain reaction (PCR) or microarrays. Among them, miR-133a (expressed in the heart and skeletal muscle) has been found to be elevated in patients with acute myocardial infarction compared to healthy individuals, as well as in elderly patients with HF (40). In a prospective analysis by Guo et al., the combination of miR-133a with NT-proBNP improved the HF diagnostic accuracy when compared to NT-proBNP alone, particularly in elderly patients (40). miR-221, which is associated with obesity, is upregulated in patients with HFpEF (40, 41). Various microRNA combinations, including miR-221, have been proposed as promising biomarkers for HF diagnosis and for differentiating HFrEF or HFpEF (41).

New biological systems, such as the apelinergic system, which includes the ligands apelin and ELABELA, have been explored for their beneficial effects in regulating endoplasmic reticulum stress (related to various cardiovascular diseases), hypertensive states, and their renoprotective effects. Plasma levels of ELABELA (detected by immunoassay) were significantly reduced in patients with HF and negatively correlated with functional class and worsening LVEF. Thus, decreased plasma ELABELA levels may serve as a novel screening biomarker for HF and could enhance diagnostic accuracy when combined with NT-proBNP (42).

HF is often associated with other comorbidities that complicate its diagnosis and worsens the prognosis. Growth-differentiation factor 15 (GDF-15), related to the inflammatory response, has emerged as a biomarker for HF and cardiovascular mortality. In obese individuals, GDF-15 plasma levels are elevated in the outpatient setting and appear to better correlate with diastolic dysfunction than NT-proBNP (43). CKD is a risk factor for the progression of cardiovascular diseases, worsening the prognosis and complicating the management of HF. NT-proBNP levels increase as glomerular filtration rate decreases, which has led to the emergence of new biomarkers for HF diagnosis in patients with CKD. A prospective analysis of 420 patients showed that soluble suppression of tumorigenesis-2 (sST2) levels was significantly higher in patients with HF. The diagnostic value of sST2 was less affected by age or renal function and suggested that it can complement NT-proBNP for HF diagnosis (44).

In the emergency setting, the accuracy of NT-proBNP for diagnosing acute decompensated HF in patients with dyspnea is markedly impaired in the presence of AF. In this context, the use of adrenomedullin (ADM) precursor molecule MR-proadrenomedullin (MR-proADM) has been proposed as an additional biomarker to NT-proBNP for the diagnosis of acute decompensated HF (45). MR-proADM is a stable peptide precursor of ADM, originating from endothelial and vascular smooth muscle cells, with vasoactive, neurohormonal and vascular endothelial protective actions. As a counteracting response to volume overload MR-proADM is released. In the endothelial cells of blood vessels it binds to specific receptors, leading to an increase in vascular integrity, and in the interstitium it causes vasodilatation (45, 46). ADM is also involved in the regulation of the renin-angiotensin-aldosterone system (RAAS) and modulation of the sympathetic nervous system in response to cardiovascular stress, such as HF (46). In a prospective analysis by Kuan et al. that included 1,107 patients with dyspnea in the emergency department, MR-proADM showed a diagnostic accuracy similar to NT-proBNP in patients without AF. However, in patients with AF, the diagnostic accuracy of NT-proBNP decreased compared to MR-proADM, suggesting that MR-proADM may improve the diagnostic strategy for acute decompensated HF in patients with elevated NT-proBNP (45). In another prospective analysis of 302 older patients presenting to the emergency department, the addition of MR-proADM or C-terminal pro-endothelin-1 (a potent vasoconstrictor primarily produced in the vascular endothelium) to NT-proBNP improved the diagnostic accuracy of acute HF (47).

Additionally, the use of other tools, such as osteopontin (a component of the matrix correlated with cardiac remodelling), has been proposed in the acute setting. Osteopontin combined with NT-proBNP emerges as a biomarker that could improve the specificity of NT-proBNP in the diagnosis of acute HF (48).

Therefore, the management of HF benefits from the identification of new biomarkers that improve diagnostic and prognostic accuracy, allowing for more effective patient care. Among those discussed, sST2, GDF-15, and MR-proADM show more advanced levels of clinical validation, although they are not yet recommended for routine diagnostic use. In contrast, microRNAs, ELABELA, and osteopontin remain at earlier stages of research, with promising but still preliminary findings. Overall, the clinical use of these emerging biomarkers remains limited by insufficient validation, lack of methodological standardization, and restricted clinical availability.

### 3.3 Imaging techniques: ultrasound imaging and other diagnostic techniques

Of the total studies resulting from this review, 47 (23.7%) articles provided information on imaging techniques aiming to improve the diagnostic process for HF. Specifically, 27 of these studies (57.4%) reported on various echocardiographic parameters and modalities, 10 (21.3%) focused on lung ultrasound (LUS), 3 (6.4%) on electrocardiography, 2 (4.3%) on bioelectrical impedance, 2 (4.3%) on cardiac magnetic resonance (CMR), 1 (2.1%) on chest radiography (CXR), 1 (2.1%) on CT pulmonary angiography, and 1 (2.1%) on phonoelectrocardiography. The most common study design was observational, with only 1 randomized clinical trial included. Regarding the type of HF classification by LVEF, 14 out of 47 (29.8%) studies included only patients with HFpEF, 3 out of 47 (6.4%) included only patients with HFrEF, and 30 out of 47 (63.8%) included both types. The high proportion of studies focusing on HFpEF seems to reflect the challenge in diagnosing this entity, despite continuous advancements in diagnostic tools and multiparametric diagnostic scores such as the Heavy, Hypertensive, Atrial Fibrillation, Pulmonary Hypertension, Elder, Filling Pressure (H_2_FPEF) or the Heart Failure Association Pre-test assessment, Echocardiography and natriuretic peptide, Functional testing, Final etiology (HFA-PEFF) scores (49, 50). In terms of the clinical presentation, a significant number of studies (19 out of 47, 40.4%) focused on the diagnosis of acute HF in the emergency department setting, highlighting the importance of an accurate and early diagnosis of HF in this context.

Echocardiography is widely recognized as a first-line imaging modality for diagnosing HF, allowing for the measurement of LVEF, as well as the quantification of other systolic function parameters, such as global longitudinal strain, and the assessment of functional and structural abnormalities (51). CMR is a less available option but holds potential value in the diagnostic evaluation of both HFpEF and HFrEF (52, 53). In patients presenting with signs and symptoms of HF, a LVEF ≤40% is sufficient to diagnose HFrEF (9). However, LVEF measurement requires trained operators and is subject to variability (54). Consequently, significant efforts have been made in recent years to develop methods for the automatic quantification of LVEF. These advances may be particularly relevant in outpatient or primary settings, where access to expert echocardiographers is often limited. For example, Hjorth-Hansen et al. assessed the feasibility and reliability of automatic software for measuring LVEF and mitral annular plane systolic excursion (MAPSE) in an outpatient cohort with suspected HF, finding only modest agreement and reliability, which suggests that further refinement and clinical validation are necessary before clinical implementation (54). Nevertheless, it is also important to consider potential inter-vendor variability among automatic software for LVEF and MAPSE quantification, and that, at least in the near future, expert review and manual quality control will remain essential to ensure measurement accuracy and clinical reliability.

For HFpEF diagnosis, echocardiography provides evidence of structural or functional abnormalities consistent with diastolic dysfunction. Traditionally, the left atrial volume index (LAVI) (55–57) and the spectral tissue Doppler-derived E/e' ratio by transthoracic Doppler echocardiography have been considered the most valid and commonly used non-invasive estimators of left ventricular filling pressures at rest (56, 58–60). More recently, left atrial strain and exercise-stress echocardiography-based measurements have demonstrated improved diagnostic performance over traditional echocardiographic measures (61–69). In this context, the advancement of AI is beginning to offer promising approaches that could facilitate improvements in the echocardiographic diagnosis of HF (70).

In the acute HF setting, LUS is an invaluable tool for the differential diagnosis of dyspnea (71–74). Nonetheless, Conangla et al. demonstrated that LUS is useful to rule-in HF in a primary care setting (75). In a meta-analysis of six studies and a total of 1,827 patients, LUS was reported to be more sensitive than CXR in detecting pulmonary edema in acute HF [0.88 (95% CI, 0.75–0.95) vs. 0.73 (95% CI, 0.70–0.76); relative sensitivity 1.20 (95% CI, 1.08–1.34); *P* < .001], while specificity was similar (71). Accordingly, in a randomized controlled trial by Pivetta et al. that included 518 patients presenting with dyspnea in the emergency department, the integration of LUS with clinical assessment was more accurate than the current diagnostic approach based on CXR and NT-proBNP (AUC 0.95 vs. 0.87, *p* < 0.01) for acute HF diagnosis (72). Notably, NT-proBNP measurement has been found to be superior to routine CXR interpretation for diagnosing or excluding acute HF, and normal CXR results should not be used to exclude HF (76). Furthermore, combined BNP and echocardiography in early hospitalization has been shown to provide useful information for patient assessment, as BNP levels correlate linearly with left ventricular systolic and diastolic dysfunction (77). Overall, the combination of LUS with echocardiographic parameters related to cardiac function, filling pressures and inferior vena cava measurements seems to have the highest accuracy for the diagnosis of HF in the acute setting (28, 60, 78–84).

Other non-ultrasound based diagnostic techniques have been evaluated for the diagnostic approach of dyspnea in the acute setting (85, 86). Sweda et al. assessed the diagnostic utility of the electrocardiogram (ECG) QRS-T angle for acute dyspnea, with a modest diagnostic accuracy [AUC 0.75 (95% CI 0.73–0.77)] that was inferior to NT-proBNP [AUC 0.93 (95% CI 0.92–0.94)] (85). Trabelsi et al. found a modest association between phonoelectrocardiography systolic time intervals and HF in emergency department patients with dyspnea, suggesting a potential role for this technique given its immediate availability (86). Techniques based on bioelectrical impedance might also be useful in this context (87, 88). In a prospective study of 290 patients admitted to the emergency department with dyspnea, the dynamic variation of impedance cardiac output with the Valsalva maneuver was the most accurate test in identifying congestive HF as the cause of dyspnea (87). In another prospective study, bioelectrical impedance analysis was found to be as useful as NT-proBNP in detecting acute decompensated HF in elderly patients with comorbidities, particularly renal failure (88). Lastly, in patients undergoing a CT pulmonary angiography that excluded a pulmonary embolism, a prospective study showed that cardiac chambers enlargement was specific for a diagnosis of HF, which might be helpful to guide treatment while waiting for confirmation (89).

In recent years, novel developments in echocardiography have extended beyond technical advancements alone. Given the increasing prevalence of HF, efficient allocation of diagnostic resources is crucial. This is particularly important in high-risk populations such as CKD and diabetic patients. In patients with CKD, the echocardiographic criteria set by the Acute Dialysis Quality Initiative were unable to effectively identify individuals at high risk for future cardiac events (90), casting doubt on the systematic utility of echocardiography in this population. In diabetic patients, Gori et al. demonstrated that an echocardiographic screening strategy guided by abnormal clinical findings and ECG data could successfully identify those at risk of developing HF (91). Moreover, a screening approach combining natriuretic peptides with handheld echocardiography may also offer an efficient method for HF detection in diabetic populations (92). Additionally, Gregers et al. reported that a normal ECG provided a 99.3% negative predictive value for excluding HFrEF in diabetic outpatients (93). Similarly, in patients referred from primary care with a suspicion of HF, the negative predictive value of a normal ECG with automated interpretation was 99% for HFrEF (94). Conversely, the usefulness of ECG to rule out HFpEF is controversial (95).

### 3.4 Artificial intelligence: a promising initiative for early HF diagnosis

The progression of HF demands advances in treatment and diagnostic techniques, leading to the rapid integration of AI. It has the potential to revolutionize medical diagnostics, treatment, risk prediction, and clinical care by interpreting wide datasets more quickly and efficiently than the human brain (96). Among the 28 studies included in this section (14.1% of the total included studies), 11 (39.3%) were selected as particularly relevant, focusing on AI-based strategies to improve HF diagnosis. These studies provided information related to the application of AI in HF screening, early detection, and risk stratification, leveraging machine learning (ML) algorithms and natural language processing (NLP) techniques. Specifically, 5 articles (45.5%) focused on applying AI to ECG-based training models, 2 (18.2%) focused on the interpretation of x-ray images using AI algorithms, 3 (27.3%) applied AI to electronic health records for early detection of HF, and 1 (9.1%) reported diagnostic AI models for decompensated HF. Most of the studies were observational (72.7%), including 5 (45.5%) prospective and 4 (36.4%) retrospective studies, as well as 1 (9.1%) systematic review and 1 (9.1%) validation study.

ML, a sub-discipline of AI, focuses on developing algorithms and models that allow computers to learn from data and improve their performance on specific tasks without explicit programming. In the medical field, ML is being increasingly applied to analyze large amounts of clinical data (images, biomedical signals, medical records, etc.), assisting physicians in decision-making and potentially improving diagnostic and treatment precision (97). In the context of HF, studies have already demonstrated the promising potential of AI and ML. Current major medical research initiatives are exploring their use to identify patients at risk of HF and to improve differential diagnosis by analyzing imaging tests (ECG, chest x-ray) and clinical history data (97–106).

One of the main characteristics of ML is its high learning capacity, enabling the identification of complex and abstract patterns (107, 108). This capability is vital for processing images, analyzing ECGs, and diagnosing diseases from complex and unstructured data (109). For example, applying AI to ECG analysis has led to AI–enabled electrocardiography (AIeECG), specifically designed for detecting left ventricular systolic dysfunction (LVSD) in primary care. Its algorithms have shown promising diagnostic accuracy in identifying LVSD [median AUC 0.90 (IQR 0.85–0.95); sensitivity 83.3% (IQR 73%–86.9%); specificity 87% (IQR 84.5%–90.9%)] and may serve as a complement to natriuretic peptide levels and echocardiograms, although further studies are needed to confirm clinical significance (98). Neural Intelligent Heart Analyzer for Heart Failure (NIHA-HF) has also been developed using an ML-based algorithm for early diagnosis and non-invasive monitoring of HF through lead I ECG analysis (99). Preliminary findings from this and other algorithms indicate that HF can be detected not only with a 12-lead ECG but also with a single-lead ECG, which can be conducted using portable devices (99, 102). Furthermore, AI and ML applications to ECG analysis have shown potential for identifying patients at risk of decompensation, which could contribute to earlier diagnosis of HF (101–103). Chiou et al. proposed an effective and accurate approach for pre-screening HFrEF using 12-lead ECG images, developed through AI-based training on 1,800 patients (101). A similar study by Bachtiger et al. showed that an AI algorithm applied to single-lead ECG could detect HFrEF using an ECG-enabled stethoscope (102). In addition, Surendra et al. developed a screening tool using an ECG-based neural network for diagnosing HF in the general population, which may be integrated into clinical routine pending further validation (103).

The application of AI to x-ray image interpretation also shows promise for early HF diagnosis, especially for HFpEF cases (104). For example, a preliminary AI model using deep learning achieved an accuracy of 82% in detecting HF on chest x-rays, suggesting potential utility to support diagnosis, particularly in primary care settings (105).

Moreover, by leveraging data from clinical records, AI may effectively predict future HF diagnoses in primary care patients, improving efficacy and precision through ongoing data processing (training) (106). Byrd et al. developed an NLP procedure that accurately identifies and labels Framingham HF diagnostic criteria in primary care clinical notes from electronic health records, potentially improving the early detection of HF (97).

Diagnostic models based on ML techniques using physiological parameters have already demonstrated high diagnostic performance for HF decompensation or chronic obstructive pulmonary disease (COPD) exacerbation. These models could offer the potential to detect clinical decompensation at an earlier phase and be implemented in routine clinical practice (110). Other models may help differentiate HFpEF from non-cardiac dyspnea or HFrEF from electronic health records, aiding in the diagnostic evaluation of patients with unexplained dyspnea (100).

More recently, investigations are being conducted to validate the usefulness of other AI-based tools able to diagnose HF or AF based on vocal cords vibration integrated with robocalls. Such research could potentially lead to massive HF screening in the community, although its clinical utility and cost-effectiveness would need to be assessed (111).

Although AI and ML applications for HF diagnosis show great potential, most remain in early development or validation stages. Their integration into clinical practice will require not only robust validation and regulatory compliance but also the development of transparent and explainable models to enhance confidence and usability.

### 3.5 Care pathways: improvement initiatives in the care process for HF diagnosis

Of the reviewed studies, 20 (10.1%) focused on processes and diagnostic circuits aimed at improving the identification of suspected HF cases. Mostly are observational, including reviews, algorithm validations, and meta-analyses, showcasing diverse approaches. Four of these articles (20%) focus on screening strategies and algorithms in primary care, 3 (15%) on high-resolution consultations and integrated diagnostic tools in the context of HFpEF, 3 (15%) on tools and protocols for diagnosis in emergency settings, 3 (15%) on specific strategies for high-risk populations such as the elderly or patients with diabetes, and 4 (20%) explore the utility of biomarkers and imaging in diagnostic processes in the context of acute diagnosis, both in emergency and primary care.

The diagnostic process for HF has evolved significantly, incorporating advanced tools and strategies to enhance early detection, particularly in high-risk populations (112). Several studies have emphasized the importance of early and accurate diagnosis to improve clinical outcomes and optimize resource allocation. One such initiative is the implementation of high-resolution outpatient consultations, which have proven effective in diagnosing HF, especially HFpEF (113, 114). By integrating clinical evaluations with echocardiography, ECG testing, and natriuretic peptide testing, these consultations have facilitated rapid and accurate diagnoses (115), resulting in timely interventions for at-risk populations, including older adults (116, 117) and those with comorbid conditions like diabetes (50, 91, 118).

Another key development is the use of screening models in primary care, particularly for patients with asymptomatic LVSD, a precursor to symptomatic HFrEF (119). Portable hand-carried ultrasound (HCU) devices have been shown to provide higher diagnostic specificity than traditional methods like ECGs or natriuretic peptides alone (56), allowing for targeted intervention and reducing unnecessary testing. Additionally, the use of decision-support tools, such as the Collaboration for the Diagnosis and Evaluation of Heart Failure (CoDE-HF) model (120), integrates clinical variables and NT-proBNP levels to guide clinicians in making more accurate and individualized diagnostic decisions, significantly enhancing diagnostic precision in acute settings.

In emergency departments, combining LUS, chest radiography, and NT-proBNP levels has improved diagnostic accuracy for acute HF, particularly in patients presenting with dyspnea (28, 76, 121). The rapid cardiothoracic ultrasound (CaTUS) protocol, which combines pulmonary and cardiac ultrasound, has demonstrated high sensitivity and specificity, supporting faster decision-making in critical settings (60). Additionally, algorithms like the HFA-PEFF score, developed for HFpEF diagnosis, have shown promise in stratifying patients based on diagnostic probability, helping clinicians decide on further invasive or non-invasive testing (49, 50, 122). In the context of HFpEF, a dedicated dyspnea clinic has proposed a standardized, stepwise diagnostic pathway (123, 124). Verwerft et al. describe a three-step algorithm for suspected HF that first estimates pre-test probability using scores such as H_2_FPEF or HFA-PEFF, natriuretic peptides, and comprehensive resting echocardiography, including left atrial strain (123, 124). Patients with low probability are referred for evaluation of non-cardiac causes of dyspnea, while those with high probability proceed to HFpEF management. Patients with intermediate probability or discordant findings undergo exercise stress echocardiography or cardiopulmonary exercise testing with echocardiography (CPET-echo). Invasive hemodynamic assessment during exercise is reserved for selected patients with persistent diagnostic uncertainty or suspicion of other mechanisms of dyspnea (124).

In conclusion, integrating these advanced diagnostic tools into clinical practice, particularly in primary and emergency care, has the potential to streamline the diagnosis of HF. By improving accuracy and reducing unnecessary testing, these initiatives contribute to better resource management and earlier interventions, ultimately improving patient outcomes. The establishment of standardized and context-specific diagnostic algorithms could facilitate the application of these tools in routine practice, ensuring consistent and efficient clinical decisions across different care settings.

A summary of the diagnostic steps and tools used to improve the HF care process is presented in Table 2 (44, 47, 98–106, 111–113).

**Table 2:** Summary of the diagnostic steps and tools used to improve the HF care process.

Step	Description
Step 1: Clinical Evaluation	Identify high-risk patients based on symptoms like dyspnea, edema, fatigue, and risk factors (age, diabetes, hypertension)
Step 2: Screening Tools	Use echocardiography, ECG, and BNP/NT-proBNP testing for initial diagnosis in primary care. Consider portable ultrasound (HCU) for screening LVSD
Step 3: Decision-Support Tools	Apply decision-support algorithms (e.g., CoDE-HF), integrating clinical data with NT-proBNP to stratify risk
Step 4: Emergency Diagnosis (AHF)	Use lung ultrasound (LUS), chest radiography, and NT-proBNP for initial assessment. Apply CaTUS protocol for rapid diagnosis
Step 5: Stratifying HFpEF	Use algorithms like HFA-PEFF to stratify HFpEF patients into low, intermediate, or high probability. In intermediate-risk or discordant findings, consider exercise stress echocardiography or CPET-echo, reserving invasive hemodynamic assessment for selected patients with persistent diagnostic uncertainty
Step 6: High-Risk Populations	For elderly and diabetic populations, use selective screening strategies (NT-proBNP and ECG) for echocardiography referrals

AHF, acute heart failure; BNP, B-type natriuretic peptide; CaTUS, cardiothoracic ultrasound; CoDE-HF, collaboration for the diagnosis and evaluation of heart failure; ECG, electrocardiogram; HCU, hand-carried ultrasound; CPET-echo, cardiopulmonary exercise testing with echocardiography; HFpEF, heart failure with preserved ejection fraction; HFA-PEFF, heart failure association pre-test assessment, echocardiography and natriuretic peptide, functional testing, final etiology; LVSD, left ventricular systolic dysfunction; LUS, lung ultrasound; NT-proBNP, N-terminal pro B-type natriuretic peptide.

## 4 Discussion

The increasing prevalence of HF and the emergence of new therapies that can be implemented even in subclinical stages with proven prognostic benefit have made early HF diagnosis a priority in both the outpatient setting and the emergency department. A significant delay in HF diagnosis may lead to unnecessary admissions, poor quality of life and higher morbidity, whereas early implementation of guideline-directed medical therapy can lower the burden of HF (125). In recent years, there has been a focus on HF screening and treatment optimization in patients considered to be at risk for developing HF, even leading to the inclusion in the 2022 AHA/ACC HF Clinical Practice Guidelines recommendations on how to manage and treat cardiovascular risk factors for patients with Stage A (“at risk”) HF, in an attempt to raise awareness and promote research in this population (126).

A strategy based on screening for natriuretic peptides and echocardiography in high-risk populations, such as patients with CKD, hypertension, or diabetes, may lead to earlier HF diagnosis. Along with natriuretic peptides, AI-enabled tools could allow earlier HF diagnosis in the outpatient setting in the near future by providing an estimated probability of HF through the analysis of readily available raw images of ECG, chest x-rays or CT scans requested for any reason, or by integrating longitudinal trends in several biomarkers from routine bloodwork, refining the available risk scores based on clinical predictors. Solutions for anticipating the development of HF should probably be individualized: for instance, in patients with pacemakers, who are usually older adults at high risk of HF, the implementation of algorithms in cardiac devices that use thoracic impedance, cardiac sounds or heart rate variability could avoid HF hospitalizations, as has been demonstrated in patients with cardiac resynchronization therapies or implantable defibrillators (127, 128). Minimally invasive techniques, such as wearable Holter monitors able to identify subclinical congestion, could also be considered to screen for worsening HF signs in very-high risk patients (129). Less invasive approaches such as multiparametric measurements obtained from a standing scale (130), or non-invasive estimation of filling pressures with multimodal wearables (131), are also being studied in the HF population and could potentially expand to patients at risk of HF.

In ambulatory patients, we suggest a high level of suspicion for HF in those with functional limitation during exertion since cardiac impairment is highly prevalent, especially when the pretest probability is high (123). The threshold for determining natriuretic peptides and referring for a comprehensive echocardiography should be low, and, when in doubt, testing during submaximal exercise should be pursued, if available in the referral center, as it frequently is able to unmask HF and avoids delays in diagnosing HF (124). The state of AI-based tools for HF have gained remarkable attention in recent years and numerous studies have developed AI or ML algorithms that can potentially increase diagnostic, prognostic and treatment performance for HF, mainly through the data analysis of ECGs, x-ray images and clinical health records. Despite these advancements, AI tools will require further evidence and robust validation for their implementation in the patient care or clinical settings (108).

However, since many of these patients who are diagnosed in their early HF stages would not have been included in most of the HF trials due to low levels of natriuretic peptides or because of no previous HF hospitalization, the symptomatic and prognostic benefit of pharmacological therapies is less well established. Therefore, caution is advised when considering drug use in this setting until further research supports this approach.

This scoping review provides a comprehensive overview of the main diagnostic and screening approaches for HF, including the integration of biomarkers, advanced imaging, AI applications, and structured care pathways, highlighting their potential relevance, particularly for patients in Stage A and B HF. However, translating these advances into routine clinical practice poses significant challenges. The application of novel tools often depends on the availability of specialized resources, expertise, and infrastructure, which may vary across regions and healthcare settings, potentially limiting their availability in primary care or general cardiology. Furthermore, the absence of globally standardized diagnostic frameworks, such as HFA-PEFF or H₂FPEF scores, limits uniform adoption and highlights the need for consensus. Moreover, AI-based tools still require robust validation and demonstration of real-world effectiveness before they can be integrated into clinical workflows. Additionally, women and other under-represented populations are often missing from the studies, raising concerns about generalizability. Finally, the evidence remains largely focused on diagnostic accuracy rather than patient-centered outcomes such as hospitalization, mortality or quality of life, underlining the need for pragmatic studies assessing real-world impact.

In conclusion, despite the substantial evidence currently available, future research should focus on evaluating the feasibility, cost-effectiveness, and real-world impact of these diagnostic strategies, while promoting standardized, accessible, and explainable approaches that can be broadly implemented to improve early detection and management of HF.

## Data Availability

The original contributions presented in the study are included in the article/Supplementary Material, further inquiries can be directed to the corresponding author.
