# Supplementary material for: Improvement initiatives in the diagnostic process of heart failure: a scoping review
Source: Front Cardiovasc Med. 2026 Jan 22;12:1681976. doi: 10.3389/fcvm.2025.1681976 (PMC12874397; doi:10.3389/fcvm.2025.1681976)
Supplement: Supplementary file 1 [file Datasheet1.docx]

Supplementary Material

# Supplementary Table 1. Search terms in PubMed and Scopus.

| Nº. | PubMed | Scopus |
| --- | --- | --- |
| #1 | ("heart failure"[title] OR "Heart Failure"[MeSH]) | (TITLE("heart failure") OR TITLE-ABS-KEY("Heart Failure")) |
| #2 | ("diagn*"[title] OR screening[title]) | (TITLE(diagn*) OR TITLE(screening)) |
| #3 | ("artificial intelligence"[title] OR "artificial intelligence"[mesh] OR "machine learning"[title] OR "neural network"[title]) | (TITLE("artificial intelligence") OR TITLE("machine learning") OR TITLE("neural network")) |
| #4 | ("imaging"[title] OR "Echocardiography" OR “echocardioscopy" OR "Magnetic Resonance"[title] OR "Computed Tomography"[title] OR (thorax[title] AND X-rays[title]) OR "ECG"[title] OR "electrocardiogram"[title]) | (TITLE("imaging") OR TITLE("Echocardiography") OR TITLE("echocardioscopy") OR TITLE("Magnetic Resonance") OR TITLE("Computed Tomography") OR (TITLE(thorax) AND TITLE("X-rays")) OR TITLE("ECG") OR TITLE("electrocardiogram")) |
| #5 | (biomarker*[title] OR BNP[title] OR “B-type natriuretic peptide”[title] OR "NT-proBNP"[title] OR "Natriuretic Peptide, Brain"[Mesh]) | (TITLE(biomarker*) OR TITLE(BNP) OR TITLE("B-type natriuretic peptide") OR TITLE("NT-proBNP")) |
| #6 | (device*[title] OR wearable*[title] OR ICD[title] OR "Implantable Cardioverter-Defibrillator"[title]) | (TITLE(device*) OR TITLE(wearable*) OR TITLE(ICD) OR TITLE("Implantable Cardioverter-Defibrillator")) |
| #7 | (“high risk”[title] OR “at risk”[title]) | (TITLE("high risk") OR TITLE("at risk")) |
| #8 | (“susp*”[title]) | (TITLE(susp*)) |
| #9 | NOT (“COVID-19”[MeSH] OR "infiltrative cardiomyopathies" OR "amyloidosis" OR "TTR" OR "sarcoidosis" OR "respiratory" OR "transplant*" OR "cardiomyopathies" OR "newborns" OR "paediatric*" OR "pediatric*" OR "nephrology" OR "cancer" OR "pulmonary hypertension" OR "aortic stenosis" OR "guideline*" OR "sleep apnea*" OR "sleep apnoea*"" ventricular assist*" OR "cirrhosis" OR "myocard*") | AND NOT (COVID-19 OR "infiltrative cardiomyopathies" OR amyloidosis OR TTR OR sarcoidosis OR respiratory OR transplant* OR cardiomyopathies OR newborns OR paediatric* OR pediatric* OR nephrology OR cancer OR "pulmonary hypertension" OR "aortic stenosis" OR guideline* OR "sleep apnea*" OR "sleep apnoea*" OR "ventricular assist*" OR cirrhosis OR myocard*) |

# Supplementary Table 2. Complete strategies.

| PubMed/Medline | | Scopus |
| --- | --- | --- |
| #1. Artificial intelligence | (("heart failure"[title] OR "Heart Failure"[MeSH]) AND ("diagn*"[title] OR screening[title]) AND ("artificial intelligence"[title] OR "artificial intelligence"[mesh] OR "machine learning"[title] OR "neural network"[title])) NOT (“COVID-19”[MeSH] OR "infiltrative cardiomyopathies" OR "amyloidosis" OR "TTR" OR "sarcoidosis" OR "respiratory" OR "transplant*" OR "cardiomyopathies" OR "newborns" OR "paediatric*" OR "pediatric*" OR "nephrology" OR "cancer" OR "pulmonary hypertension" OR "aortic stenosis" OR "guideline*" OR "sleep apnea*" OR "sleep apnoea*"" ventricular assist*" OR "cirrhosis" OR "myocard*") | (TITLE("heart failure") OR TITLE-ABS-KEY("Heart Failure")) AND (TITLE(diagn*) OR TITLE(screening)) AND (TITLE("artificial intelligence") OR TITLE("machine learning") OR TITLE("neural network")) AND NOT (COVID-19 OR "infiltrative cardiomyopathies" OR amyloidosis OR TTR OR sarcoidosis OR respiratory OR transplant* OR cardiomyopathies OR newborns OR paediatric* OR pediatric* OR nephrology OR cancer OR "pulmonary hypertension" OR "aortic stenosis" OR guideline* OR "sleep apnea*" OR "sleep apnoea*" OR "ventricular assist*" OR cirrhosis OR myocard*) |
| #2. Imaging techniques | (("heart failure"[title] OR "Heart Failure"[MeSH]) AND ("diagn*"[title] OR screening[title]) AND ("imaging"[title] OR "Echocardiography" OR “echocardioscopy" OR "Magnetic Resonance"[title] OR "Computed Tomography"[title] OR (thorax[title] AND X-rays[title]) OR "ECG"[title] OR "electrocardiogram"[title])) NOT (“COVID-19”[MeSH] OR "infiltrative cardiomyopathies" OR "amyloidosis" OR "TTR" OR "sarcoidosis" OR "respiratory" OR "transplant*" OR "cardiomyopathies" OR "newborns" OR "paediatric*" OR "pediatric*" OR "nephrology" OR "cancer" OR "pulmonary hypertension" OR "aortic stenosis" OR "guideline*" OR "sleep apnea*" OR "sleep apnoea*"" ventricular assist*" OR "cirrhosis" OR "myocard*") | (TITLE("heart failure") OR TITLE-ABS-KEY("Heart Failure")) AND (TITLE(diagn*) OR TITLE(screening)) AND (TITLE("imaging") OR TITLE("Echocardiography") OR TITLE("echocardioscopy") OR TITLE("Magnetic Resonance") OR TITLE("Computed Tomography") OR (TITLE(thorax) AND TITLE("X-rays")) OR TITLE("ECG") OR TITLE("electrocardiogram")) AND NOT (COVID-19 OR "infiltrative cardiomyopathies" OR amyloidosis OR TTR OR sarcoidosis OR respiratory OR transplant* OR cardiomyopathies OR newborns OR paediatric* OR pediatric* OR nephrology OR cancer OR "pulmonary hypertension" OR "aortic stenosis" OR guideline* OR "sleep apnea*" OR "sleep apnoea*" OR "ventricular assist*" OR cirrhosis OR myocard*) |
| #3. Biomarkers | (("heart failure"[title] OR "Heart Failure"[MeSH]) AND ("diagn*"[title] OR screening[title]) AND (biomarker*[title] OR BNP[title] OR “B-type natriuretic peptide”[title] OR "NT-proBNP"[title] OR "Natriuretic Peptide, Brain"[Mesh])) NOT (“COVID-19”[MeSH] OR "infiltrative cardiomyopathies" OR "amyloidosis" OR "TTR" OR "sarcoidosis" OR "respiratory" OR "transplant*" OR "cardiomyopathies" OR "newborns" OR "paediatric*" OR "pediatric*" OR "nephrology" OR "cancer" OR "pulmonary hypertension" OR "aortic stenosis" OR "guideline*" OR "sleep apnea*" OR "sleep apnoea*"" ventricular assist*" OR "cirrhosis" OR "myocard*") | (TITLE("heart failure") OR TITLE-ABS-KEY("Heart Failure")) AND (TITLE(diagn*) OR TITLE(screening)) AND (TITLE(biomarker*) OR TITLE(BNP) OR TITLE("B-type natriuretic peptide") OR TITLE("NT-proBNP")) AND NOT (COVID-19 OR "infiltrative cardiomyopathies" OR amyloidosis OR TTR OR sarcoidosis OR respiratory OR transplant* OR cardiomyopathies OR newborns OR paediatric* OR pediatric* OR nephrology OR cancer OR "pulmonary hypertension" OR "aortic stenosis" OR guideline* OR "sleep apnea*" OR "sleep apnoea*" OR "ventricular assist*" OR cirrhosis OR myocard*) |
| #4. Devices | (("heart failure"[title] OR "Heart Failure"[MeSH]) AND ("diagn*"[title] OR screening[title]) AND (device*[title] OR wearable*[title] OR ICD[title] OR "Implantable Cardioverter-Defibrillator"[title])) NOT (“COVID-19”[MeSH] OR "infiltrative cardiomyopathies" OR "amyloidosis" OR "TTR" OR "sarcoidosis" OR "respiratory" OR "transplant*" OR "cardiomyopathies" OR "newborns" OR "paediatric*" OR "pediatric*" OR "nephrology" OR "cancer" OR "pulmonary hypertension" OR "aortic stenosis" OR "guideline*" OR "sleep apnea*" OR "sleep apnoea*"" ventricular assist*" OR "cirrhosis" OR "myocard*") | (TITLE("heart failure") OR TITLE-ABS-KEY("Heart Failure")) AND (TITLE(diagn*) OR TITLE(screening)) AND (TITLE(device*) OR TITLE(wearable*) OR TITLE(ICD) OR TITLE("Implantable Cardioverter-Defibrillator")) AND NOT (COVID-19 OR "infiltrative cardiomyopathies" OR amyloidosis OR TTR OR sarcoidosis OR respiratory OR transplant* OR cardiomyopathies OR newborns OR paediatric* OR pediatric* OR nephrology OR cancer OR "pulmonary hypertension" OR "aortic stenosis" OR guideline* OR "sleep apnea*" OR "sleep apnoea*" OR "ventricular assist*" OR cirrhosis OR myocard*) |
| #5. High risk of HF | (("heart failure"[title] OR "Heart Failure"[MeSH]) AND ("diagn*"[title] OR screening[title]) AND (“high risk”[title] OR “at risk”[title])) NOT (“COVID-19”[MeSH] OR "infiltrative cardiomyopathies" OR "amyloidosis" OR "TTR" OR "sarcoidosis" OR "respiratory" OR "transplant*" OR "cardiomyopathies" OR "newborns" OR "paediatric*" OR "pediatric*" OR "nephrology" OR "cancer" OR "pulmonary hypertension" OR "aortic stenosis" OR "guideline*" OR "sleep apnea*" OR "sleep apnoea*"" ventricular assist*" OR "cirrhosis" OR "myocard*") | (TITLE("heart failure") OR TITLE-ABS-KEY("Heart Failure")) AND (TITLE(diagn*) OR TITLE(screening)) AND (TITLE("high risk") OR TITLE("at risk")) AND NOT (COVID-19 OR "infiltrative cardiomyopathies" OR amyloidosis OR TTR OR sarcoidosis OR respiratory OR transplant* OR cardiomyopathies OR newborns OR paediatric* OR pediatric* OR nephrology OR cancer OR "pulmonary hypertension" OR "aortic stenosis" OR guideline* OR "sleep apnea*" OR "sleep apnoea*" OR "ventricular assist*" OR cirrhosis OR myocard*) |
| #6. Clinical suspicion of HF | (("heart failure"[title] OR "Heart Failure"[MeSH]) AND ("diagn*"[title] OR screening[title]) AND (“susp*”[title])) NOT (“COVID-19”[MeSH] OR "infiltrative cardiomyopathies" OR "amyloidosis" OR "TTR" OR "sarcoidosis" OR "respiratory" OR "transplant*" OR "cardiomyopathies" OR "newborns" OR "paediatric*" OR "pediatric*" OR "nephrology" OR "cancer" OR "pulmonary hypertension" OR "aortic stenosis" OR "guideline*" OR "sleep apnea*" OR "sleep apnoea*"" ventricular assist*" OR "cirrhosis" OR "myocard*") | (TITLE("heart failure") OR TITLE-ABS-KEY("Heart Failure")) AND (TITLE(diagn*) OR TITLE(screening)) AND (TITLE(susp*)) AND NOT (COVID-19 OR "infiltrative cardiomyopathies" OR amyloidosis OR TTR OR sarcoidosis OR respiratory OR transplant* OR cardiomyopathies OR newborns OR paediatric* OR pediatric* OR nephrology OR cancer OR "pulmonary hypertension" OR "aortic stenosis" OR guideline* OR "sleep apnea*" OR "sleep apnoea*" OR "ventricular assist*" OR cirrhosis OR myocard*) |

# Supplementary Table 3. Evidence extraction table summarizing included studies.

| **Authors** | **Year** | **Study design** | **Sample size (*n*)** | **HF phenotype** | **Clinical setting** | **Intervention/ Strategy** | **Outcomes** | **Notes and limitations** |
| --- | --- | --- | --- | --- | --- | --- | --- | --- |
| A. A. Martinez-Rumayor et al. | 2010 | Comparative study | 599 | Suspected acute HF | Emergency Department | Biomarker vs imaging (NT-proBNP vs CXR) | NT-proBNP superior to CXR for HF detection | CXR should not be used to exclude HF |
| A. Baessler et al. | 2012 | Observational study | 207 | LVDD with possible HF | Outpatient | Biomarker enhancement (GDF-15 vs NT-proBNP) | AUC 0.74 vs 0.56 for LVDD; P < 0.0001 | GDF-15 correlates better in obese patients |
| A. Barandiarán Aizpurua et al. | 2020 | Validation study | 687 | HFpEF | Outpatient/  Ambulatory | Validation of HFA-PEFF score (step 2) | Diagnostic accuracy 0.90; specificity 93%; PPV 98% | 36% intermediate category needing further testing |
| A. Celik et al. | 2023 | Diagnostic accuracy study | 10,100 | HF (54.9% HFpEF) | Outpatient | AI-assisted imaging (CXR interpretation) | PPV for diagnosing HF: 77%, NPV: 91% | Patients >45 y; 1.8% flagged; 54.9% HFpEF among new diagnoses |
| A. Ceriello et al. | 2023 | Screening study | 259 | HF risk | Diabetes outpatient | POCT NT-proBNP screening | 60.6% (n = 157) NT-proBNP <125 pg/mL and 39.4% (n = 102) ≥125 pg/mL | Practical for routine screening in T2D + hypertension |
| A. Gohar et al. | 2019 | Model development | 1,371 | High-risk HF | Primary Care | Sex-specific logistic regression with NT-proBNP | C-statistics improved with NT-proBNP (up to 0.80) | Validated for early detection of LVDD/HFpEF |
| A. Iacovoni et al. | 2013 | NA | NA | Asymptomatic LVSD/Stage B | Primary Care | BNP + Framingham Risk Score | Useful for screening high-risk patients | Letter to the Editor DAVID-BERG study |
| A. Javeed et al. | 2022 | Systematic review | NA | General heart disease, including chronic HF | Not specified | ML/data mining diagnostic systems using clinical data, ECG, images | Affordable, efficient, and reliable heart disease detection | Reviews multiple modalities; focus not only on HF |
| A. Joharimoghadam et al. | 2017 | Pilot study | 70 | Chronic HF | Inpatient/ Outpatient cardiology | Salivary and plasma BNP measurement | Salivary BNP higher in HF patients | Small sample; preliminary; needs validation |
| A. K. Hjorth-Hansen et al. | 2022 | Clinical trial (diagnostic/ feasibility study) | 166 | Suspected HF | Outpatient | Hand-held ultrasound with autoEF/autoMAPSE | Feasibility 50–91%; intra-rater ICC 0.51–0.85; inter-rater ICC 0.35–0.51 | Modest feasibility; not ready for clinical practice |
| A. M. Maw et al. | 2019 | Systematic review and meta-analysis | 1,827 | Acute decompensated HF | Various (dyspnea patients) | Lung ultrasound vs chest X-ray | LUS more sensitive than CXR (0.88 vs 0.73); specificity similar (0.90); relative sensitivity ratio 1.2 | Only 6 studies; supports LUS as adjunct for early pulmonary edema detection |
| A. M. Yeung et al. | 2023 | Consensus report | Not applicable | Stage A and Stage B HF in diabetic population | Outpatient | Standardized early HF screening | Annual BNP/NT-proBNP; echo if abnormal | Consensus-based; no new empirical data |
| A. Maisel et al. | 2010 | Prospective multicenter study | 1,641 | Acute HF | Emergency Department | MR-proANP vs BNP; MR-proADM prognosis | MR-proANP noninferior to BNP; MR-proADM predicts 90-day mortality | Supports added value of novel biomarkers; MR-proADM adds prognostic value |
| A. P. Nikolova et al. | 2018 | Cohort study | 52 | HFpEF | Outpatient/ Ambulatory | Plasma cBIN1-derived score (CS) vs NT-proBNP | AUC 0.98 vs 0.93; HR 3.8 for 1-year CV hospitalization | Promising biomarker for early HFpEF detection; needs further validation |
| A. Palazzuoli et al. | 2016 | Observational study | 310 | Acute HF | Emergency Department/ Early hospitalization | BNP correlation with cardiac function | Correlates with EF, diastolic dysfunction, TAPSE, PAPs | Supports BNP as marker of global cardiac dysfunction in early acute HF |
| A. Sekma et al. | 2021 | Prospective diagnostic study | 184 | Acute HF (HFpEF/HFrEF) | Emergency Department | ΔCO after sublingual nitroglycerin | Sensitivity 80%; specificity 44%; AUC 0.70 | Simple bedside tool; moderate accuracy; needs further validation |
| A. Squizzato et al. | 2021 | Systematic review and meta-analysis | 591 | Acute HF | Emergency Department | IVC collapsibility index (ultrasound) | Sensitivity 79%, specificity 82%; suboptimal as stand-alone test | May aid diagnosis when combined with other tools; not sufficient alone |
| A. Yamanoğlu et al. | 2015 | Prospective observational study | 74 | Acute HF (vs pulmonary dyspnea) | Emergency Department / ICU | Sonographic IVC diameter (B-mode) | Sensitivity 84.4%; specificity 92.9%; +LR 11.8; −LR 0.16 | Rapid, noninvasive; best among IVC methods reported |
| A. Zegard et al. | 2023 | Retrospective study | 654 | Suspected HF (all LVEF) | Primary to secondary care referral | NT-proBNP >400 ng/L | Predicted HF LVEF<40% OR 10.2; HF any LVEF OR 6.13; correctly identified 54.5% | NT-proBNP is better for general cardiac disease rather than HF per se |
| B. Vaes et al. | 2010 | Cross-sectional diagnostic study | 80 | Chronic HF | Community-based (nonagenarians) | NT-proBNP measurement compared with 2D echo | Cut-off 269.5 pg/mL: sens 85%, NPV 77%, AUC 0.75 | High NPV; limited to well-functioning very elderly |
| C. Boyang et al. | 2022 | Bioinformatic / computational study | NA | HF (type not specified) | Genomic datasets | Random forest and artificial neural network using gene expression | Identified 6 key genes; validated in 2 datasets | Model potentially useful for cardiac biopsy-based HF prediction; further validation required |
| C. Carette et al. | 2024 | Prospective screening study | 1,506 | Subclinical HF in obesity | Ambulatory nutrition clinic | BNP ≥35 pg/mL + echo assessment | BNP ≥35 pg/mL associated with remodeling, LV mass ↑, atrial changes, higher PAP | Identifies subclinical remodeling; supports BNP screening in high-risk obesity |
| C. Gálvez-Barrón et al. | 2023 | Prospective observational trial | 135 | Decompensated HF (and/or COPD) | Hospital and home follow-up | 6MWT with continuous HR/SpO2 + ML models | LR: sens 80.8%, spec 86.3%, acc 83.6%; SVM: sens 81.7%, spec 85%, acc 82.8% | Preliminary models; require further validation |
| C. J. Taylor et al. | 2017 | Prospective observational diagnostic validation study | 304 | Suspected HF | Primary Care | Clinical decision rule ± NT-proBNP | CDR+NT-proBNP: sens 90.4%, spec 45.5%; NT-proBNP <125 pg/mL: sens 94.2%, spec 49%; <400 pg/mL: sens 76.9%, spec 91.5% | Low threshold improves detection; high threshold may under-refer |
| C. J. Taylor et al. | 2023 | Diagnostic accuracy study | 229,580 | Chronic HF | Primary Care | NT-proBNP at ESC ≥125 pg/mL & NICE ≥400 pg/mL | ESC: sens 94.6%, spec 50%, NPV 98.9%; NICE: sens 81.7%, spec 80.3%, NPV 97.7% | Reliable rule-out; threshold choice depends on healthcare priorities |
| C. Liu et al. | 2024 | Observational monocentric study | 335 | HF (all LVEF, including HFpEF) | Hospital-based | Plasma ELABELA and Apelin measurement | ELABELA: AUC 0.835, sens 62.5%, spec 95.9%; Apelin: AUC 0.673, sens 66.2%, spec 67.2% | ELABELA may be novel screening biomarker; combined BNP+ELABELA could improve accuracy |
| C. R. Goyder et al. | 2023 | Systematic review and meta-analysis | 26,565 | LVSD | Ambulatory (general and high-risk) | NT-proBNP and BNP measurement | NT-proBNP cut-off 311 pg/mL: sens 0.74, spec 0.85; BNP 49 pg/mL: sens 0.68, spec 0.81 | NP screening may accurately detect LVSD in high-risk; prospective studies needed |
| C. R. Olsen et al. | 2020 | Narrative review | NA | General HF | NA | Overview of machine learning and AI applications | Describes current ML uses for diagnosis, phenotyping, prognosis | Educational; no new empirical data |
| C. S. Ma et al. | 2022 | Prospective diagnostic study | 389 | HFpEF | Ambulatory / Outpatient (risk population) | Novel LA strain parameters (LASr, stiffness, filling index) | AUC 0.756–0.843 for elevated LVEDP & HFpEF discrimination | Supports potential incorporation of LA strain into diagnostics |
| C. S. Son et al. | 2012 | Diagnostic modeling study | NA | Chronic HF (with dyspnea) | Emergency Department | Rough sets (RS) and logistic regression (LR) models using key features | RS: acc 97.5%, sens 97.2%, spec 97.7%, AUC 97.5%; LR: acc 88.7%, AUC 88.8% | RS better than LR (p<0.01); highlights role of Pro-BNP |
| C. Watson et al. | 2016 | Observational study | 1,368 | Stage B HF (asymptomatic) | Primary Care | BNP in diabetic vs non-diabetic patients | AUC 0.75 (DM), 0.77 (non-DM); 80% sens requires 5-ng/L lower threshold in DM | Slight effect of diabetes on BNP; clinically minor |
| C. Xie et al. | 2023 | Systematic review and meta-analysis | 345 samples | Acute HF | Emergency Department | Lung ultrasound vs CT and echocardiogram | LUS accuracy comparable to CT; superior to echo; high sens & spec y | Few studies; heterogeneity; possible publication bias |
| D. J. Whellan et al. | 2013 | Retrospective analysis | 166 | HF (post-hospitalization) | Outpatient | CRT-D device follow-up (impedance, AF, pacing, HR variability) | High-risk group HR 25.4 for 30-day readmission; device parameters identified at-risk patients | Small cohort; limited to CRT-D; further validation needed |
| D. K. Kayembe et al. | 2023 | Experimental study | 132 | Subclinical HF | Laboratory / experimental | VaSera® non-invasive vascular stiffness device (ET + PEP) to estimate Ees/Ea | ICC 0.71; 4 measurements sufficient; CCC 0.99; Ees/Ea 1.5 [1.2–1.9] | Healthy volunteers only; preliminary; not tested in HF patients |
| D. Kumar et al. | 2021 | ML framework development | NA | HF (type not specified) | Dataset analysis | PKI-secured IoT framework (CDF-DI) with Random Forest to identify cardiac disease features | RF: survival prediction accuracy 96%; gender 94%; age group 96%; key features: follow-up months, SC, EF, CPK, platelets | Secondary dataset; no real-world clinical validation |
| D. M. Li et al. | 2021 | Diagnostic accuracy study | 180 | HFrEF vs HFpEF | Outpatient | miR-208a + NT-proBNP measurement | miR-208a + NT-proBNP: AUC 0.83 vs 0.73 (NT-proBNP alone); sens 68%, spec 90.2% for HFrEF diagnosis | Small sample; preliminary; needs validation in larger cohorts |
| D. Robaei et al. | 2011 | Randomized controlled study | 68 | Suspected HF | Emergency Department | NT-proBNP measurement provided to ED physicians | Diagnostic uncertainty reduced from 66% → 18%; diagnostic accuracy improved 53% → 71% | Small sample; single-center; preliminary; focused on physician decision-making rather than patient outcomes |
| D. Saura et al. | 2015 | Cross-sectional study | 269 | Suspected HF | Hospital (admitted with dyspnea) | Echocardiography detection of systolic aortic regurgitation (SAR) | High specificity 99.4%; sensitivity 7.5%; PPV 88.9%; positive LR 11.85 | SAR rare (3.3%); low sensitivity; may serve as “black box” predictor; preliminary |
| E. Dal Canto et al. | 2022 | Systematic review and meta-analysis | 353 (20 studies) | HFpEF/ LVDD | Various, Cardiology | Echocardiography + multivariable models | Multivariable AUC 0.95; LA strain AUC 0.83; exercise E/e' ↑ sensitivity 90% | High bias risk; supports multivariable approach |
| E. E. Unlüer et al. | 2012 | Diagnostic accuracy study | 69 | Diastolic dysfunction / suspected HF | Emergency Department | Bedside echocardiography by trained ED physicians | Sens 89%, Spec 80%, Accuracy 87% vs cardiologist report | Small sample; single center; short training; ED setting |
| E. Lanzarone et al. | 2023 | Validation study | 73 | HFpEF | Cardiology / Outpatient | HFA-PEFF algorithm (echo + natriuretic peptides ± DSE) vs invasive RHC | Sens 45–46% (step 2–3); Spec 88–100%; step 2 sensitivity could rise to 60% if threshold >3 | Small sample; limited sensitivity; non-invasive algorithm may underclassify HFpEF |
| E. Pivetta et al. | 2019 | Randomized controlled trial | 518 | ADHF / acute dyspnoea | Emergency Department | Lung ultrasound + clinical assessment vs CXR/NT-proBNP | LUS + clinical: AUC 0.95; CXR/NT-proBNP: AUC 0.87; diagnostic errors reduced 7.98 vs 2.42 per 100 patients | Two-center study; ED setting; limited to acute dyspnoea |
| E. Won et al. | 2015 | Retrospective chart review | 83 | New-onset HFrEF | Cardiology | CMR with LGE, cine, FPP for IC vs NIC | LGE C-stat 0.85; LGE + cine: Spec 87% IC, Spec 94% NIC; FPP added no value | Small sample; retrospective; coronary angiography within 6 months; single-center |
| F. C. Bennis et al. | 2022 | Retrospective ML study | 8,543 | Incident HF | Primary Care | ML model using demographics + codes | Best model AUC 0.772; NNS reduced from 14.11 → 5.99 per true positive | Retrospective; age ≥70; generalizability limited to GP data; prediction 1 year before diagnosis |
| F. Gouzi et al. | 2021 | Systematic review and meta-analysis | 1,009 HF / 833 HC | HF (type not specified) | Various / Diagnostic studies | Exhaled breath acetone (ExA) measurement | ExA 1.89× higher in HF; correlated with NYHA and BNP | High heterogeneity; promising but needs standardization |
| F. M. Russell et al. | 2015 | Prospective observational study | 99 | ADHF / acute dyspnea | Emergency Department | 12-view lung & cardiac ultrasound (LuCUS) | Sens 83%, Spec 83%, +LR 4.8, −LR 0.20; 47% had management changes; accuracy +20% vs clinical gestalt | Small sample; single-center; operator-dependent |
| F. S. Gaborit et al. | 2020 | Prospective study | 399 | HF/ asymptomatic LVSD | Outpatient | MR-proANP vs NT-proBNP measurement | MR-proANP AUC 0.886, NT-proBNP AUC 0.910; both associated with HF; NT-proBNP adds more diagnostic info | Non-acute setting; moderate sample; mostly high-risk outpatients |
| F. S. Gharehchopogh et al. | 2011 | Case study | 40 | HF (type not specified) | Health center/  hospital | Neural network-based data mining for decision support | NN predicted 85% of test cases correctly | Very small sample; single center; limited generalizability |
| F. Wang et al. | 2010 | Observational study | 208 | Stage B HF / early HF | Outpatient | Plasma GDF-15 measurement | Correlated with HF stage (r=0.804); AUC 0.873 for stage B HF | Moderate sample; single-center; screening potential only |
| F. Yasmin et al. | 2021 | Narrative review | NA | HF (type not specified) | Various / Diagnostic and clinical care | AI applications: neural networks, decision trees, logistic regression for HF detection | NN accuracy 85%; AI aids imaging analysis, ECG interpretation, risk prediction, and management optimization | No new data; review only; heterogeneous studies; general overview of AI in HF |
| G. D. Sanna et al. | 2021 | Comprehensive review | NA | HF / HFpEF / subclinical HF | Various / Echocardiography | Global longitudinal strain (GLS) analysis | Useful for diagnosis and prognosis across HF phenotypes | Review only; no new data; variability across vendors; cutoff values not standardized |
| G. Guidi et al. | 2012 | System development | NA | HF (type not specified) | Clinical / Decision support | AI-based CAD system with 4 AI techniques | Comparative accuracy across models | No patient numbers; simulated or retrospective data; generalizability limited |
| G. Li et al. | 2018 | Observational study | NA | Acute HF | Outpatient / Hospital | Circulating miR-302 family measurement | miR-302b-3p AUC 0.87; correlated with NT-proBNP | Sample size not specified; single-center; preliminary biomarker study |
| G. Murtagh et al. | 2012 | Observational study | 814 | Preclinical ventricular dysfunction / LVSD | Outpatient / Primary care | BNP alone (20, 50, 100 pg/mL) ± ECG; Doppler echo confirmation | Sensitivity BNP alone: 88–45%; specificity 46–90%; many false positives had LVDD; optimal screen (BNP ≥50 or abnormal ECG) sensitivity 80%, specificity 72% | Single-center; observational; focuses on screening; economic impact not assessed |
| H. Alawieh et al. | 2019 | Narrative review | NA | HF (type not specified) | Various / Point-of-care | BNP and NT-proBNP biosensor technologies for POC testing | Improved diagnostic accuracy (~80%) vs traditional methods | Review only; no new patient data; focus on technology and feasibility rather than clinical trial results |
| H. Chen et al. | 2021 | Systematic review and meta-analysis | 19 studies | Chronic HFpEF | Various / outpatient | Diagnostic biomarkers: BNP, NT-proBNP, Galectin-3, ST2 | Sensitivity: BNP 0.79, NT-proBNP 0.70, Gal-3 0.76, ST2 0.64; Specificity: NT-proBNP 0.88, BNP 0.80, Gal-3 0.80, ST2 0.60 | Limited by small number of studies for Gal-3 and ST2; heterogeneity across studies |
| H. Ma et al. | 2023 | Comparative analysis | 420 | Chronic HF | Hospital / outpatient | Biomarkers: NT-proBNP, sST2, individually and combined | Optimal cut-offs: NT-proBNP total 1280 pg/mL, non-CKD 481 pg/mL, CKD 3314 pg/mL; sST2 ~29-31 ng/mL; combined model improved diagnostic accuracy | sST2 less affected by renal function; combined biomarkers superior; CKD impacts NT-proBNP |
| H. Røsjø et al. | 2015 | Observational study | 309 | Acute HF | Emergency Department | NT-proBNP measurement in standard vs. deglycosylated plasma | AUC 0.871 vs 0.852; better mortality prediction | Deglycosylation improves NT-proBNP utility; single-center ED study |
| H. Yan et al. | 2017 | Systematic review and meta-analysis | 10 studies | HF (mixed phenotypes) | Various | Circulating miRNAs (overall, miR-423-5p) ± BNP | miR-423-5p: Sens 0.81 / Spec 0.67; total miRNAs: Sens 0.74 / Spec 0.69; BNP: SROC 0.93; miRNAs + BNP: slight improvement | miRNAs alone less accurate than BNP; miR-423-5p most promising; high heterogeneity |
| H. Yang et al. | 2016 | Prospective cohort | 410 | Stage B / at-risk HF | Community | Echocardiography (GLS, LV mass, LA volume, E/e'), clinical evaluation | Predictors of incident HF: GLS, LV mass, LAE, LVH, E/e'; GLS added incremental info; event rate 104/1000 person-years | Excluded valve disease, AF, reduced EF; short follow-up (14 mo); only elderly |
| H. Zhang et al. | 2022 | Experimental / Computational study | NA | Chronic HF | Experimental | Korotkoff sound classification via BO-SVM | BO-SVM: Acc 85%, Se 85.3%, Sp 84.6% for CHF classification | Small experimental dataset; feasibility study; requires further clinical validation |
| I. A. Fazal et al. | 2015 | Observational cohort | 208 | HFrEF and HFpEF | Heart failure clinic | NT-proBNP referral thresholds (Newcastle, NICE, ESC), echocardiography | Newcastle thresholds more sensitive; NICE missed 8 HF; ESC similar yield but more referrals | Median age 77.5 y, mostly women; small sample; follow-up ~17 mo |
| I. Malhamé et al. | 2019 | Retrospective study | 60 | HF (pregnant/ postpartum) | Obstetrics | BNP measurement + expert adjudication | Sens 95%, Spec 62% at BNP 111 pg/mL; PPV 2.5, NPV 0.1 | Small cohort; single center; mostly preeclampsia patients; mostly postpartum |
| I. Oudejans et al. | 2012 | Validation study | 203 | HF (older adults ≥70) | Geriatric outpatient | Diagnostic algorithms (ESC, NICE, NL, Mant) vs outcome panel | ESC NT-proBNP <400 pg/mL: 52% referred, 78% HF among referred, 10% false negatives | Small sample; older adults only; guideline algorithms compared |
| I. Trabelsi et al. | 2020 | Prospective diagnostic study | 855 | HF (all with dyspnea) | Emergency department | Phonoelectrocardiography STIs (EMAT, LVET, EMAT/LVET) ± BNP | EMAT/LVET ≥40%: Sens 72%, Spec 88%, c-statistic 0.78; improves likelihood ratio when combined with BNP | Intermediate BNP subgroup; correlation with LVEF moderate; single-center |
| J. A. Schaub et al. | 2015 | Systematic review and meta-analysis | 4,287 | Acute decompensated HF | Various (incl..renal dysfunction subgroups) | NT-proBNP in renal vs non-renal patients | Dx AUC 0.66–0.89 (renal dysf), 0.72–0.95 (normal); Prognosis RR mortality ~3.2 | Renal dysfunction raises cutoff; heterogeneity partly due to HF/CAD |
| J. B. Du et al. | 2012 | Observational study | 156 | Chronic HF | Hospitalized patients | Plasma BNP ± serum T3 | BNP alone: Sens 90.8%, Spec 95.5%; T3 negatively correlated with severity; combination improves prognostic evaluation | Single-center; NYHA-based classification; prognostic value mainly for T3 |
| J. C. Kelder et al. | 2011 | Cross-sectional diagnostic study | 721 | New-onset HF | Outpatient/ HF clinics | History + physical exam items ± NT-proBNP | c-statistic 0.83 (history/PE alone), 0.86 (with NT-proBNP); external validation c-statistics 0.95 and 0.88 | NT-proBNP strongest additive diagnostic tool; study limited to specialized HF outpatient clinics |
| J. Cho et al. | 2021 | Retrospective cohort study | 24,211 | HFrEF | Hospital | AI algorithm using 12-lead and single-lead ECG for HFrEF detection | 12-lead ECG: AUC 0.913-0.961; Single-lead ECG: AUC 0.874-0.929 | Multicenter; interpretable AI; wearable single-lead applicability demonstrated |
| J. Cho et al. | 2023 | Comparative diagnostic study | 160 | HF vs non-HF | Laboratory | Comparison of 3 NT-proBNP assays (Atellica IM, Alere, Elecsys proBNP II) | All assays acceptable; Elecsys had higher specificity and positive likelihood ratio; AUCs comparable | Small sample size; in vitro comparison; clinical outcomes not longitudinal |
| J. Draper et al. | 2018 | Observational study | 413 | HFrEF 40.6%, HFpEF 31.1%, HFmrEF 28.2% | Tertiary hospital | NT-proBNP measurement for HF diagnosis | Overall AUC 0.734; AUC reduced by 7.5% in patients >80; lowest NT-proBNP in HF >80: 466 pg/ml | NT-proBNP less accurate in patients >80; suggests threshold modification for ruling out HF in elderly |
| J. Eckstein et al. | 2012 | Prospective cohort | 632 | Acute HF | Emergency Department | MR-proANP vs NT-proBNP for AHF diagnosis in AF vs sinus rhythm | AUC: MR-proANP 0.90, NT-proBNP 0.89 in AF; higher cut-offs needed in AF; MR-proANP predicted 1-year mortality | Rhythm affects peptide levels; diagnostic cut-offs need adjustment |
| J. J. Atherton | 2010 | Review | NA | Asymptomatic LV systolic dysfunction | Primary care | Clinical scores, ECG, natriuretic peptides, hand-carried echocardiography | Hand-carried echo provides favorable diagnostic performance for screening | Review; questions remain on target population, operator, training, and screening frequency |
| J. L. Martindale et al. | 2016 | Systematic review and meta-analysis | 117 studies | Acute HF | Emergency Department | Clinical assessment + CXR + BNP/NT-proBNP + LUS + bedside echo | LUS and echo: LR+ 4.1–7.4; BNP <100 pg/mL, NT-proBNP <300 pg/mL: LR– 0.09–0.11; B-lines LR– 0.16 | Wide prevalence range (29–79%); heterogeneity; pooled data; diagnostic thresholds vary |
| J. Li et al. | 2018 | Observational study | 219 | HFrEF, HFmrEF | Cardiology | Plasma GDF-15 ± NT-proBNP measurement | GDF-15 ↑ with HF stage; HFrEF> HFmrEF; GDF-15+NT-proBNP AUC 0.905 vs NT-proBNP 0.869; GDF-15 correlates with remodeling | Single-center; 1-year follow-up; no prognostic value |
| J. M. Verdú et al. | 2012 | Diagnostic accuracy study | 220 | HFrEF and HFpEF | Primary Care | NT-proBNP point-of-care measurement | Best cut-off 280 pg/mL; AUC 0.94; 6 HF patients (<12%) <400 pg/mL; could avoid 67% of echocardiograms | Single center; older population; low N of HF events |
| J. Öhman et al. | 2019 | Prospective study | 100 | Acute HF | Emergency Department (tertiary care) | CaTUS (E/e’ + LUS) | Sens 100%, Spec 95.8%, AUC 0.979; better than E/e' or LUS alone | Single center; small sample; reference standard included BNP and echo |
| J. Song et al. | 2011 | Observational study | 160 | HF (All NYHA classes I–IV) | Clinical/  Outpatient | Urinary BNP measurement vs plasma BNP | Urinary BNP correlated with plasma BNP, NYHA class, LVEF; predicted post-treatment events; noninvasive | Single-center; not randomized; no sensitivity/specificity reported |
| J. Wang et al. | 2023 | Observational study | 94 | Chronic HF | Clinical/  Outpatient | Measurement of serum lncRNA LUCAT1 and BNP | LUCAT1 low in CHF; combined AUC better than individual. Prognostic value confirmed | Single-center; small sample; follow-up duration not specified; no numeric sensitivity/specificity provided |
| K. Cui et al. | 2018 | Comparative biomarker study | 190 | HFpEF and HFrEF | Clinical/  Outpatient | Measurement of plasma MR-proANP and NT-proBNP | MR-proANP higher diagnostic accuracy for HFpEF than NT-proBNP (AUC 0.844 vs 0.518, P<0.001) | Small sample; single-center; levels correlated with NYHA class |
| K. Hebert et al. | 2010 | Screening study | 145 | Stage B HF | Outpatient | BNP measurement + handheld echocardiography | BNP AUC 0.77; echo interpretation reliability κ=1.0. BNP may serve as a screening tool for stage B HF | Did not correlate BNP with diastolic dysfunction; single population (diabetics); limited generalizability |
| K. K. Lee et al. | 2022 | Meta-analysis and modeling | 10,369 | Suspected acute HF | Emergency Department | CoDE-HF combining NT-proBNP and clinical data | NPV 94.6%; rule-in PPV varied by age (61–80%); CoDE-HF AUC 0.846–0.925, Brier 0.099–0.130 | Performance varied across key subgroups; tool validated internationally |
| K. L. Anderson et al. | 2013 | Prospective cohort | 101 | Acute decompensated HF | Emergency Department | Cardiac, IVC, and lung ultrasound | Combined US: specificity 100%, sensitivity 36% | Single-center; small sample; combination of all 3 US measures decreased sensitivity but maximized specificity |
| K. Ng et al. | 2016 | Retrospective modeling study | 1,684 | Incident HF | Primary Care | Machine learning prediction using longitudinal EHR data | Performance improved with data diversity and density | No external validation; Guidelines for data requirements proposed |
| K. Shaikh et al. | 2011 | Observational, Cross-sectional study | 100 | Acute HF | Emergency Department | Plasma NT-proBNP measurement, CXR, echocardiography | Sensitivity 100%, specificity 86% (age-adjusted cutoffs); NPV 100% (age-independent cutoff 300 pg/ml) | Single-center; small sample; no external validation |
| K. Surendra et al. | 2023 | Observational study | 5,299 | General population | Outpatient | CNN on digital ECGs to detect HF | AUC 0.75; sensitivity 0.67; specificity 0.69 | Population-based; HF prevalence 6%; single-city study |
| K. Untersteller et al. | 2018 | Validation study | 472 | HF in CKD | Outpatient/ Nephrology | ADQI echocardiographic criteria | 66% met HF criteria; 87% of these were not hospitalized for HF over 4.3 ± 2.0 yrs | Overdiagnosis risk; poor prognostic value |
| K. V. Bunting et al. | 2021 | Subanalysis of RCT | 160 | HF with AF | Outpatient / hospital | Index-beat vs conventional averaging for LVEF, GLS, E/e' | Improved reproducibility and efficiency | Applicable to AF patients; single-center data, limited to echocardiographic measures |
| L. Chen et al. | 2021 | Observational study | 260 | HFpEF | Not specified | Measurement of microalbuminuria (MAU) and NT-proBNP; ROC analysis | MAU: AUC 0.83 (sens 72.5%, spec 82%); NT-proBNP: AUC 0.88 (sens 82%, spec 73.8%); Combined: AUC 0.91 | MAU levels not correlated with HF severity; improves diagnostic accuracy when combined with NT-proBNP |
| L. Chen et al. | 2021 | Deep learning-based study | NA | HF (early detection in elderly) | Hospital-based | CBAM-CNN attention convolutional neural network on ECG signals | Effectively classifies ECG signals; performance improved with signal preprocessing | Sensitive to noise; real-world validation mentioned but details limited |
| L. Conangla et al. | 2020 | Prospective diagnostic study | 162 | Suspected HF | Primary Care | Lung ultrasound (LUS) with criteria C1 and C2; combined with Framingham, ECG, ± NT-proBNP | LUS-C2: specificity 0.99, PPV 0.92; AUC 0.90 when combined with other criteria vs 0.84 without; reclassified 1/3 of patients in absence of NT-proBNP | Small, single-center; primarily elderly women; NT-proBNP not always available; needs external validation |
| L. D. Liastuti et al. | 2022 | Cross-sectional diagnostic study | 138 | HF (type not specified) | Inpatient | LIFES model (LSTM on echo videos) | Accuracy: A2C 92.96%, A4C 90.62%, PLAX 88.28% | Feasible and fast; best performance with A2C view; further external validation needed |
| L. Guo et al. | 2014 | Prospective cohort study | 177 | Acute dyspnea due to CHF or APE | Emergency Department | NT-proBNP measurement via ECLIA + TTE | NT-proBNP cut-offs discriminate CHF vs APE; sens/spec reported | Requires confirmatory imaging; single-center, small sample |
| L. Han et al. | 2017 | Pilot study | 117 | Decompensated chronic HF | Inpatient | Plasma relaxin and BNP measurement; combined assessment | Relaxin sensitivity 82.7%, specificity 55.6%; combined relaxin+BNP improved diagnostic accuracy vs BNP alone | Small sample; preliminary data; nonlinear correlation with cardiac function |
| L. J. Boonman-de Winter et al. | 2015 | Diagnostic model development study | 581 | Suspected HF in type 2 diabetes | Primary Care | Clinical screening model (history, symptoms, signs ± ECG, NT-proBNP) | C-statistic 0.80–0.86; sensitivity 70.8%; specificity 79%; NPV 87.6%; PPV 56.4% | Useful to pre-select for echocardiography; modest reclassification gain (0.06) adding ECG/NT-proBNP |
| L. J. Gula et al. | 2014 | Validation study | 1,224 | Chronic HF (CRT-D / ICD patients) | Ambulatory / Device-based monitoring | Implantable integrated diagnostics algorithm combining rhythm, HR, activity, and fluid data | HF hospitalization risk: low 0.21%, medium 0.66%, high 2.61% per month; HR 2.9 (medium) and 10.7 (high) vs low risk | ID score correlated with HF hospitalization and symptoms; potential adjunct for early detection and management |
| L. Li et al. | 2023 | Observational study | 338 | HF vs non-HF | Laboratory | NT-proBNP assays comparison (Elecsys proBNP II vs SuperFlex NT-proBNP; ± glycosidase treatment) | ROC-AUC 0.930–0.943; total NT-proBNP similar to non-glycosylated fraction | Total NT-proBNP may improve sensitivity in asymptomatic patients |
| L. O'Halloran et al. | 2020 | Pilot study | 230 | Suspected HF | Emergency Department | CT pulmonary angiography assessment for HF | Specificity ~100% for LV, LA, RV enlargement; CTPA closely matched echocardiography | Only 24/230 (10.4%) had HF; small sample; pilot study |
| L. V. Bjerkén et al. | 2023 | Systematic review | 15 studies | LVSD | Various | AI-enabled ECG (AIeECG) for opportunistic LVSD screening | Median AUC 0.90; sensitivity 83.3%; specificity 87% | Heterogeneous LVEF thresholds; promising as adjunct to NP and echocardiography |
| M. A. Msolli et al. | 2021 | Prospective study | 290 | Undifferentiated dyspnea; HF vs non-HF | Emergency Department | Bioimpedance cardiac output under sitting position (SP), leg raising (LR), and Valsalva maneuver (VM) | VM: sensitivity 79%, specificity 60%, likelihood ratios 1.97/0.36; AUC SP 0.62, LR 0.63, VM 0.70 | VM had independent diagnostic value; SP and LR performed poorly; HF diagnosis based on BNP, echo, and clinical assessment |
| M. Averina et al. | 2022 | Observational study | 1,936 | Subclinical HF | General population | AUC < 0.75 for both biomarkers; Biomarker-based screening (age/sex-specific NT-proBNP and hs-troponin T cut-offs) | higher specificity with age/sex cut-offs vs standard; low sensitivity limits screening utility | Good for ruling in HF, not for ruling out; suboptimal sensitivity, especially in mild or younger cases |
| M. Behnes et al. | 2014 | Diagnostic and prognostic study | 212 | Acute HF (HFrEF, HFpEF) | Emergency Department | Combined biomarker testing (CTGF + NT-proBNP) | CTGF + NT-proBNP non-inferior to NT-proBNP alone; improved accuracy 82%, specificity 83%, PPV 66%, NRI +0.11 | CTGF adds value for acute decompensation; CTGF correlates with higher HF severity; no prognostic value at 1 or 5 years |
| M. Behnes et al. | 2013 | Diagnostic and prognostic study | 401 | Acute congestive HF | Emergency Department | Combined biomarker testing (Osteopontin + NT-proBNP) | Improved diagnostic accuracy (76%, specificity 74%, NRI +0.10, p = 0.0001); Osteopontin independently predicted mortality and rehospitalization at 1 and 5 years | stronger prognostic role than NT-proBNP for aCHF-related rehospitalization |
| M. Bombelli et al. | 2015 | Diagnostic study | 895 | HF (type not specified) | Emergency Department | NT-proBNP cut-off optimization for HF diagnosis | Optimal thresholds: 980 pg/mL (Sn 0.95; NPV 0.90) and 5340 pg/mL (Sp 0.85; PPV 0.76) | Good diagnostic performance in very elderly; eGFR, Ht, CRP did not affect accuracy; ~42% initially in diagnostic uncertainty area |
| M. C. T. Gregers et al. | 2022 | Observational and diagnostic study | 772 | HF in type 2 diabetes | Hospital (secondary care) | ECG screening for HF detection and prognosis | NPV >99% for ruling out HFrEF/ALVSD | Supports ECG use to rule out HF in T2D |
| M. F. Seronde et al. | 2013 | Diagnostic study | 710 | Acute HF | Emergency Department | BNP, proBNP, NT-proBNP, MR-proANP | Diagnostic AUC: BNP 0.973, MR-proANP 0.901; MR-proANP best for 5-year prognosis | All NPs similar for diagnosis; MR-proANP superior for long-term prognosis |
| M. Gori et al. | 2017 | Prospective cohort study | 219 | HF in diabetes | Outpatient | Clinical + ECG + NT-proBNP + Echo | Adding ECG improved prediction (C-stat 0.75 vs 0.70, p<0.05); NT-proBNP did not (0.72, p=0.20); | ECG adds value; NT-proBNP less predictive alone |
| M. Guazzi | 2016 | Narrative review | NA | HFpEF | Not specified | Stress echocardiography + CPET | Opens diagnostic window for HFpEF | Conceptual; no quantitative data |
| M. Guo et al. | 2018 | Diagnostic study | 125 | HF (type not specified) | Hospital-based | Biomarker evaluation (circulating miR-133a, miR-221 ± NT-proBNP) | miR-133a + NT-proBNP AUC 0.975; improved over NT-proBNP alone | miRNAs unaffected by age, BMI, renal or metabolic factors; specific to elderly population |
| M. Landolfo et al. | 2024 | Cross-sectional diagnostic study | 148 | HF (HFrEF, HFmrEF, HFpEF) | Inpatient | NT-proBNP + LUS + TTE | NT-proBNP inversely correlated with LVEF (cut-off ≥9531 pg/mL): OR 2.5; combined markers: OR 4.3 | NT-proBNP and LUS useful for HF confirmation but poor for HFrEF discrimination |
| M. M. Alqezweeni et al. | 2024 | Diagnostic modeling study | 132 | Chronic HF | Laboratory | Neural network classification of blood tensiometric data | 99% classification accuracy | laboratory-based simulation study; not yet validated clinically |
| M. Smeets et al. | 2016 | Diagnostic accuracy study | 365 | Non-acute HF | Primary Care | Comparison of four diagnostic algorithms | ESC: sensitivity 92%, referral rate 71%; Oudejans: specificity 73%, referral 36% | Trade-off between sensitivity and specificity driven by NT-proBNP cut-offs |
| M. V. Carlino et al. | 2018 | Diagnostic accuracy study | 102 | Acute HF | Emergency Department | Lung-heart-IVC ultrasound with PUD | 96% accuracy with combined approach | Small sample; single-center |
| N. Farajidavar et al. | 2022 | Diagnostic modeling study | 1,585 + 269 | HFpEF | Inpatient and Outpatient | Multi-modal EHR data with gradient boosting | AUROC 0.90; precision 74% | Requires validation; retrospective design |
| N. Génot et al. | 2015 | Prospective diagnostic study | 77 | Acute HF | Emergency Department | Bioelectrical impedance vector analysis (BIVA) vs BNP and echocardiography | Ra cutoff 39Ω: sensitivity 67%, specificity 76%, AUC 0.76 | Correlated with BNP; no added value over BNP alone |
| N. Ghosh; H. Haddad | 2011 | Review | NA | HF (type not specified) | General | ANP and MR-proANP | MR-proANP useful in BNP gray zone; prognostic for mortality | Incremental value over BNP in specific contexts |
| N. Hammoudi et al. | 2017 | Prospective diagnostic study | 60 | HFpEF | Inpatient | Low-level exercise echocardiography vs catheterization | Septal E/e' at 25W: AUC 0.79; in cardiac disease patients: AUC 0.96 | Not reliable in patients without coronary disease or LV abnormalities |
| N. Kozhuharov et al. | 2021 | Prospective diagnostic study | 1,083 | Acute HF | Emergency Department | Cardiac myosin-binding protein C (cMyC) vs hs-cTnT and NT-proBNP | cMyC AUC 0.81; NT-proBNP AUC 0.91; cMyC predicts mortality (HR 2.19) | cMyC useful for triage; not independent predictor in validated models |
| N. Kozhuharov et al. | 2019 | Prospective diagnostic study | 2,038 | Acute HF | Emergency Department | NT-proBNP cut-off concentrations | Age-adjusted cut-offs improve specificity | Validated in large cohort; supports clinical use |
| N. Kozhuharov et al. | 2022 | Prospective diagnostic study | 2,053 | Acute HF | Emergency Department | NT-proBNP cut-offs adjusted for obesity | Sensitivity increased to 98.2%; specificity 76.5% | Reduces gray zone; improves rule-in/out accuracy |
| N. L'Hermitte et al. | 2024 | Prospective diagnostic study | 166 | Acute HF | Emergency Department | Ultrasound-based algorithm including deceleration time (DT) | AUC 0.91; Sensitivity 87%; Specificity 95% | No undetermined diagnoses; strong performance |
| N. Nishii et al. | 2015 | Observational study | 195 | HF (type not specified) | Primary care | OptiVol alert + intrathoracic impedance (ITI) | ITI decrease ≥4% improves BNP-based diagnosis | BNP alone not sufficient; ITI adds diagnostic value |
| N. Saxena; L. S. Maurya | 2019 | Diagnostic modeling study | NA | Congestive HF | General | Decision Tree algorithm | Improved accuracy over previous models | Supports cardiologists; efficient execution |
| Health Quality Ontario | 2021 | Health Technology Assessment | 12 studies | Suspected HF | Emergency Department and Community Care | Use of BNP and NT-proBNP testing for suspected HF | High sensitivity (80–94% BNP, 86–96% NT-proBNP) and cost-effectiveness; reduced hospital stay in ED | No impact on mortality or readmission; limited evidence in community setting |
| O. Taheri et al. | 2023 | Prospective diagnostic study | 238 | Acute HF | Emergency Department | NT-proBNP, hs-cTnI, ST2, Gal-3, CD146 measured alone and in combination | NT-proBNP alone: AUC 0.72; best combined: AUC 0.73 | No improvement over NT-proBNP alone |
| P. Almeida et al. | 2018 | Case-control study | 195 | HFpEF / HFrEF | Inpatient | Echocardiography with tissue Doppler imaging; BNP, GLS, E/E' ratio, LAVI | LAVI showed highest diagnostic accuracy (AUC 0.90) | Small sample size; stroke patients as controls |
| P. Bachtiger et al. | 2022 | Prospective multicenter study | 1,050 | HFrEF | Outpatient | AI-ECG applied to single-lead ECG via ECG-enabled stethoscope; multiple anatomical positions | Pulmonary + handheld positions: AUROC 0.91, sensitivity 91.9%, specificity 80.2% | Effective point-of-care tool; validated multicentre |
| P. Bahrmann et al. | 2015 | Prospective diagnostic study | 302 | Acute HF | Emergency Department | NT-proBNP alone or combined with MR-proADM, CT-proET-1, MR-proANP, Copeptin-us | Adding MR-proADM or CT-proET-1 to NT-proBNP improved diagnostic accuracy: C-index 0.84–0.86 vs 0.81 | Older population (≥70 yrs); multimarker combinations beyond MR-proADM or CT-proET-1 not beneficial |
| P. Gil Martínez et al. | 2016 | Prospective observational study | 96 | Acute decompensated HF | Emergency Department | NT-proBNP vs inferior vena cava ultrasound (IVCu) vs bioelectrical impedance analysis (BIA) | IVCu and BIA comparable to NT-proBNP for ADHF diagnosis; AUC 0.90 for max IVC, 0.93 for min IVC | Small sample; NT-proBNP influenced by renal impairment, IVCu and BIA not |
| P. Nazerian et al. | 2010 | Diagnostic accuracy study | 145 | Acute LVHF | Emergency Department | Emergency Doppler echocardiography (EDecho) by emergency physicians | Restrictive pattern: sensitivity 82%, specificity 90%, accuracy 75% | More accurate than NT-proBNP and Boston criteria |
| Q. Han et al. | 2024 | Observational case-control study | 198 | Chronic HF | Outpatient / Hospital | Serum miR-320a-3p measurement by qRT-PCR | miR-320a-3p elevated in CHF; AUC 0.866 for diagnosis; high expression associated with poor 4-year prognosis | Independent prognostic factor; mechanistic pathways explored only via bioinformatics |
| R. A. Mohamed et al. | 2023 | Observational retrospective study | 248 | De novo systolic HF (HFrEF) | Primary Care referrals | ECG assessment (automatic vs cardiologist interpretation) | Abnormal ECG associated with reduced LVEF; NPV 99%; adding ECG to risk model increased AUC from 0.72 to 0.79 | ECG effective gatekeeper for echocardiography |
| R. Andrea et al. | 2013 | Observational descriptive study | 143 | New-onset HF: HFpEF and HFREF | Primary Care (one-stop clinic) | Multimodal assessment: cardiologist evaluation, ECG, CXR, BNP, echocardiography | BNP cut-off 60.12 pg/mL: Sensitivity 83%, Specificity 84%, AUC 0.898 | High prevalence of HFpEF; predictors differ by phenotype |
| R. C. Rimbas et al. | 2022 | Prospective observational study | 125 | HFpEF / preHF with diastolic dysfunction | Outpatient | Conventional echocardiography + 2D speckle tracking echocardiography (LA strain analysis) | LA pump function (SR_CT) < -1.66/s plus GS and sPAP best identified HFpEF; AUC 0.76; LA strain parameters correlated with NTproBNP | LA pump function best differentiates HFpEF from preHF; ; findings need external validation |
| R. Chen et al. | 2019 | Observational/Modeling study | 34,502 (4370 HF cases) | Incident HF | Primary Care | Machine learning vs recurrent neural network for HF prediction using EHR data | RNN performance superior under most conditions; improved with greater data quantity | Optimal results require sufficient training data; data diversity affects performance |
| R. E. Harskamp et al. | 2023 | Retrospective diagnostic study | 15,234 | Suspected HF | Primary Care | BNP vs NT-proBNP; guideline vs personalized thresholds | NT-proBNP AUROC 0.899 vs BNP 0.859; personalized cut-offs modestly improved accuracy | NT-proBNP more accurate than BNP; personalized thresholds only modestly better |
| R. F. Kievit et al. | 2018 | Diagnostic IPD meta-analysis | 1,941 | Suspected HF | Community / Primary care | Clinical prediction model using age, IHD history, exertional dyspnea, BMI, apex beat ± NT-proBNP | C-statistic 0.70–0.82 (model), 0.89 with NT-proBNP; good calibration | Model identifies candidates for echocardiography; needs prospective clinical implementation |
| R. H. Christenson et al. | 2010 | Observational study | 685 | Suspected decompensated HF | Community | BNP and NT-proBNP measured across BMI categories | NT-proBNP better predictor of mortality in normal BMI | BMI affects biomarker levels; cut-offs may need adjustment |
| R. J. Byrd et al. | 2014 | NLP-based retrospective study | NA | Suspected HF | Primary Care | NLP system to extract Framingham HF criteria from EHR notes | High precision and recall (F-score >0.9) | System may improve early detection; requires adaptation for other settings |
| R. Parvan et al. | 2022 | Systematic review and meta-analysis | 29 studies | HFrEF and HFpEF | Various | Circulating microRNA panels | HFrEF: Sensitivity 0.85, Specificity 0.88, AUC 0.91; HFpEF: Sensitivity 0.82, Specificity 0.61, AUC 0.79 | miRNA panels show potential additive value to BNP/NT-proBNP; can help rule out HF; conventional biomarkers still superior; further validation needed |
| R. Ro et al. | 2011 | Diagnostic comparison study | 250 | Suspected HF | Emergency Department | POC BNP measurement: Triage vs i-STAT vs central lab | AUC: Triage 0.95, i-STAT 0.98; i-STAT faster (median 9 min) vs Triage (19 min); device failures rare | Both accurate; i-STAT faster but less specific |
| R. S. Small et al. | 2014 | Retrospective analysis | 175 | HF (type not specified) | Hospital / post-discharge | Device-derived diagnostic criteria at discharge: intrathoracic impedance, AF burden, CRT pacing, night HR | HFR rates at 30 days: ≥2 criteria 28%, 1 criterion 16%, 0 criteria 7%; ≥2 criteria HR 5.0 for 30-day HFR | Device criteria at discharge identify high-risk patients |
| R. Sarzani et al. | 2016 | Prospective observational study | 403 | Suspected HF | Internal Medicine / Geriatrics | NT-proBNP measurement (<300 pg/mL rule-out; ≥1800 pg/mL rule-in) | NT-proBNP ≥1800 pg/ml associated with in-hospital mortality | Very high prevalence of underlying HF; NT-proBNP useful for identifying cardiac involvement |
| R. Sweda et al. | 2020 | Prospective diagnostic study | 1,915 | Suspected ADHF | Emergency Department | QRS-T angle vs NT-proBNP and hs-TnT | QRS-T AUC 0.75; NT-proBNP AUC 0.93 | QRS-T angle useful for risk stratification |
| R. T. Campbell et al. | 2020 | Proteomic analysis | 829 | HF (acute and chronic) | Ambulatory/ Hospital | Urinary proteomic classifier HF1 vs BNP | HF1 AUC 0.94; BNP AUC 0.98; HF1 + BNP AUC 0.99, NRI 0.67; HF1 less prognostic than BNP | HF1 adds incremental diagnostic info to BNP; poorer prognostic value |
| S. A. Hill et al. | 2014 | Systematic review | 76 studies | Suspected HF | Emergency Department | BNP and NT-proBNP measuremen | BNP: Sensitivity 95%, Specificity 55; NT-proBNP: Sensitivity 96%, Specificity 55 | Performs well to rule out HF, less well to rule in; Effect of age, gender, renal function on cutpoints unclear; |
| S. Albani et al. | 2024 | Narrative review | NA | HFpEF | Outpatient / acute care | Biomarkers, imaging, score-based algorithms, invasive evaluation | Summarizes strengths and weaknesses of diagnostic tests for HFpEF; guidance for clinical decision-making | No quantitative outcomes; narrative synthesis |
| S. Arques | 2021 | Narrative review | NA | HFpEF | Outpatient / acute care | E/e' ratio via Doppler echocardiography | Valid for assessing LV pressures at rest/exercise | No new primary data; emphasizes routine reporting of E/e' in HFpEF evaluation |
| S. Arques et al. | 2021 | Prospective diagnostic study | 58 | HFpEF | Emergency Department | Transthoracic Doppler echocardiography measuring peak mitral E-wave velocity (E), E/e', E/(e'x s') | E >85 cm/s: Sensitivity 90%, Specificity 93%, AUC 0.95 | Simple and effective for non-expert operators |
| S. Arques et al. | 2010 | Prospective diagnostic study | 26 | Diastolic HF | Outpatient | Plasma BNP measurement; reference standard: invasive LVEDP | BNP predictive of DHF (p=0.03); cut-off 31 pg/mL: Sensitivity 67%, Specificity 73%; AUC 0.76 (0.55–0.9), p=0.007 | Useful in young patients with isolated dyspnea |
| S. Arques et al. | 2010 | Observational diagnostic study | 28 | Diastolic HF | Outpatient | Transthoracic echocardiography measuring left atrial volume index (LAVi) | LAVi >38 ml/m²: AUC 0.84, Sensitivity 60%, Specificity 100%; standard cut-off 34 ml/m²: Sensitivity 70%, Specificity 88% | LAVi predictive of DHF; validated against catheterization |
| S. Coiro et al. | 2023 | Prospective case-control study | 170 | HFpEF | Outpatient | Exercise lung ultrasound (LUS) measuring B-lines; submaximal ESE and maximal CET | Peak B-lines >5: Sensitivity 93.4%, Specificity 97.5%; Change B-lines >3: Sensitivity 94.7%, Specificity 87.5% | Adds diagnostic accuracy to HFpEF scores and BNP; effective across protocols and experience levels |
| S. D. Feng et al. | 2017 | Observational study | 65 | Acute left HF  ± AF | Outpatient | Plasma BNP and beta-endorphin (β-EP) measurement; LVEF by ECG | BNP: sens 93.5%, spec 81.3%, AUC 0.921; β-EP: sens 80.5%, spec 78.6%, AUC 0.697; combined: sens 94.1%, spec 83.5%, AUC 0.604–0.979 | Combined biomarkers improve early diagnosis; correlations with LVEF |
| S. Di Somma et al. | 2014 | Observational diagnostic study | 381 | Acute HF | Emergency Department | Bioelectrical impedance vector analysis (BIVA) measuring hydration index (HI) | AUC 0.87, Sensitivity 90%, Specificity 54%; NRI for diagnosis 77%, prognosis 45% | BIVA adds value in BNP grey-zone; may improve patient management and 30-day prognosis; overall BIVA did not improve BNP accuracy outside grey-zone |
| S. J. Backhaus et al. | 2024 | Prospective diagnostic study | 68 | HFpEF vs non-cardiac dyspnoea | Outpatient / Cardiology | Rest and exercise-stress echocardiography + LA compliance measurement | LA compliance at rest: AUC 0.87; predicted CV hospitalization (HR 2.83, 95% CI 1.70–4.74, p<0.001) | 4-year follow-up; LA compliance can be integrated into routine echo |
| S. Norman et al. | 2023 | Prospective observational study | 236 | Suspected HF | Inpatient / Hospital | NT-proBNP-guided TTE triage (≥900 high-priority, 300–899 intermediate, <300 low/outpatient) | Reduced LOS (9.97 vs 13.87 days, p<0.00); no 30-day deaths in low/intermediate; safe outpatient discharge for low NT-proBNP | NT-proBNP triage reduces hospital stay; safe for low-risk patients |
| S. P. Shaik et al. | 2024 | Literature review | NA | HFpEF | Outpatient | Evaluation of emerging biomarkers (ST2, Galectin-3, microRNAs) | No biomarker superior to NP for diagnosis or prognosis; ST2 and Gal-3 promising | No primary data; potential for phenotype-specific biomarker profiles |
| S. R. Meisel et al. | 2012 | Randomized controlled study | 470 | Acutely decompensated HF | Emergency Department | NT-proBNP testing at ED admission | Improved discharge diagnosis (unblinded 74.5% vs blinded 61.9%); NT-proBNP allowed risk stratification | No impact on length of stay or 2-year mortality |
| S. Sartini et al. | 2017 | Prospective observational study | 236 | Acute HF | Emergency Department | Lung ultrasound, chest X-ray, NT-proBNP, individually and combined | Lung US: sens 57.7%, spec 88%; CXR: sens 74.5%, spec 86.3%; NT-proBNP: sens 97.6%, spec 27.6%; combined CXR + US: sens 84.7%, spec 77.7%, NPV 87.1% | No single best diagnostic test identified; stepwise approach suggested (CXR + US first, then NT-proBNP if negative) |
| S. Singh et al. | 2020 | Narrative review | NA | HFpEF and HFrEF | Outpatient | NP measurement (BNP, NT-proBNP) with BMI considerations | Diagnostic accuracy of NPs reduced in overweight/obese patients; prognostic value preserved across BMI classes | Limited evidence for severe obesity (BMI >40 kg/m²); lower NP thresholds and clinical context recommended for diagnosis |
| S. van Doorn et al. | 2018 | Meta-analysis of individual data | 1,941 | HF in AF | Community | NT-proBNP screening for HF | NT-proBNP cut-off 125 pg/mL: sens 93%, spec 35%, PPV 51%, NPV 86% | High HF prevalence in AF; echocardiography suggested as preferred screening method |
| S. W. Rabkin | 2021 | Systematic review | Not specified (13 studies) | HFpEF vs HFrEF | Not specified | GDF-15, Galectin-3, sST2 vs BNP | GDF-15, Gal-3, sST2 elevated in HFpEF vs controls; BNP better at differentiating HFpEF from HFrEF | Combined indices with BNP may improve differentiation |
| S. Zhang | 2023 | Retrospective, observational study | 166 | HFpEF | Cardiology | CMR strain analysis (LV, RV, LA) | Combined LV strain AUC 0.858 for diagnosis (sens 0.713, spec 0.875); prognostic AUC 0.722 for endpoint events | Individual strains less predictive; Combined strain analysis more useful than individual parameters |
| T. Breidthardt et al. | 2022 | Secondary analysis of multicenter study | 2,485 | Acute HF | Emergency Department | Assessment of diurnal variation in BNP, NT-proBNP, MR-proANP | Higher diagnostic accuracy in evening/nighttime for BNP/NT-proBNP | Diurnal variation confirmed in 44 stable individuals; MR-proANP unaffected |
| T. D. Trippel | 2021 | Observational study | 1,386 | HFpEF risk / incident HFpEF | Outpatient | Galectin-3 measurement | AUC 0.71; sensitivity 0.61, specificity 0.73; Gal-3 predicted incident HFpEF, adjusted all-cause mortality, and composite CV hospitalization + death | Diagnostic value statistically significant but clinical relevance debatable |
| T. G. Papadopoulos | 2021 | ML model development | 422 | HF (type not specified) | Cardiology | Deep learning (AE, DNN, RBM, Autoencoder) using clinical, demographic, echo & lab data | Best model (Autoencoder + DNN): accuracy 91.7%, sensitivity 90.7%, specificity 92.3%, | Retrospective dataset; High performance; mixed features (clinical + imaging + labs) |
| T. Harada | 2023 | Diagnostic accuracy study | 487 | HFpEF | Outpatient / Cardiology | Echocardiographic left atrial compliance during exercise (LA reservoir strain / E/e′) | AUC 0.87; superior to E/e’ ratio; incremental value over resting compliance | Validated in two protocols; enhances HFpEF diagnosis in dyspnea |
| T. Matsumoto | 2020 | Diagnostic model development (AI/deep learning) | 638 | HF (type not specified) | Hospital (NIH database) | Deep learning on chest X-rays images | Accuracy 82%; heatmap visualization supports interpretability | Promising tool; based on NIH dataset and cardiologist relabeling |
| T. Mueller et al. | 2016 | Prospective diagnostic and prognostic study | 251 | Acute HF | Emergency Department | Galectin-3, soluble ST2 (sST2), BNP | Diagnostic: BNP AUC 0.92 > sST2 0.63 > Galectin-3 0.57. Prognostic: All similar for 1-year mortality | BNP superior for diagnosis; all similar for prognosis |
| T. W. Churchill | 2021 | Validation study | 156 | HFpEF | Hospital / Cardiology | Evaluation of HFA-PEFF algorithm and H₂FPEF score vs invasive hemodynamic HFpEF definition | HFA-PEFF AUC 0.73; sensitivity 72%, specificity 91%; H₂FPEF AUC 0.74; sensitivity 31%, specificity 92% | Both associated with exercise impairment; letter format |
| T. Weber | 2013 | Diagnostic accuracy study | 136 | HFpEF | Hospital / Cardiology | Tissue Doppler echocardiography ± arterial pulsatile function measures (PWV, aoPP, bPP, Pb) | AUCs: aoPP 0.851, aPWV 0.867; improved AUC with combined measures (AUC 0.875–0.901) | Pulsatile measures complement TDE; improved classification accuracy |
| U. Siebert | 2021 | Economic evaluation | NA | Suspected acute HF | Emergency Department | NT-proBNP-supported strategy vs clinical assessment alone | Reduced hospitalizations, echo use, ICU admissions; cost savings $2,337/visit | Model-based analysis; robust across sensitivity analyses; Medicare perspective |
| V. Homar et al. | 2021 | Scoping/ systematic review | NA | HF screening in elderly | Nursing home | Natriuretic peptide biomarkers (NT-proBNP, BNP) for HF screening | NT-proBNP higher in HF (2409 pg/mL vs 1074 pg/mL); cut-offs 230–760 pg/mL | Current cut-offs may be too low for nursing home use |
| W. B. Horton et al. | 2024 | Screening study | 1,664 | Stage B HF | Outpatient/ Diabetes type 1 | Prevalence and distribution of NT- proBNP and BNP testing | Median NT- proBNP value: 97 pg/mL; BNP and NT- proBNP greater in older patients and those with serum creatinine ≥1.50 mg/dL | First study to quantify HF screening by NP distributions in a real- world T1D cohort. |
| W. S. Kuan et al. | 2020 | Diagnostic performance comparison | 1,107 | Acute decompensated HF (with or without AF) | Emergency Department | Biomarker measurement: MR-proADM, NT-proBNP, hs-cTnT | MR-proADM: AUC 0.83 non-AF, 0.76 AF; accuracy 73.3% in AF, superior to NT-proBNP and hs-cTnT | MR-proADM superior in AF; consistent across sites |
| X. X. Shuai et al. | 2011 | Diagnostic validation study | 334 | HFpEF | Outpatient / Cardiology | Echocardiographic parameters: lateral E/e', LAVI, Ard-Ad | Combined strategy sensitivity 77%, specificity 81% for HFpEF | Validated in two cohorts; simple and accurate strategy |
| Y. A. Chiou et al. | 2021 | AI model development | 1,800 | HFrEF/ HFmrEF | Outpatient / Cardiology | 2D-CNN on 12-lead ECG; V6 lead highest accuracy | Highest accuracy 0.93, sensitivity 0.97, specificity 0.89 for lead V6 | Combined V5–V6 leads provided optimal diagnostic index; applicability to real-world screening needs further evaluation |
| Y. Chen | 2024 | Research Letter | NA | HF (type not specified) | General | NIHA-HF model using Lead-I ECG | Potential to enhance early detection; only requires lead I; can be integrated with any portable ECG devices | Conceptual model; preliminary findings |
| Y. Jin et al. | 2021 | Case-control diagnostic study | 236 | Acute HF | Hospital / Cardiology | Biomarker measurement: miR-214, BNP, NT-proBNP, sST2 | AUCs: miR-214 0.913, BNP 0.836, NT-proBNP 0.849, sST2 0.855 | All biomarkers significantly higher in AHF vs controls; levels correlate with NYHA class |
| Y. N. V. Reddy et al. | 2018 | Score development and validation | 514 | HFpEF | Hospital | Clinical and echocardiographic variables combined into H_2_FPEF score | AUC 0.841 derivation; 0.886 validation; odds of HFpEF doubled per 1-unit increase | Outperforms consensus algorithm; guides further testing |
| Y. Pan et al. | 2017 | Diagnostic model study | 436 | Suspected chronic HF (HFrEF, HFmrEF, HFpEF) | Outpatient | NT-proBNP measurement with corrected diagnostic formula including age, BMI, GFR, AF, sex | Improved diagnostic accuracy: AUC 0.955; sensitivity 94.2%; specificity 86.7% | Improved over standard NT-proBNP cut-off; model derived and validated internally |
| Y. Saito et al. | 2023 | Diagnostic accuracy study | 254 | HFpEF | Hospital outpatient | Exercise stress echocardiography with simultaneous cardiopulmonary exercise testing (CPET) and expired gas analysis | Modest diagnostic value in differentiating HFpEF from noncardiac dyspnea (AUC <0.61) | Substantial overlap between HFpEF and NCD; modest diagnostic utility |
| Y. Tamaki et al. | 2023 | Diagnostic accuracy study | 121 | Acute HF | Emergency  Department | Echocardiography: VMT score ± lung ultrasonography (LUS) | VMT score: Sensitivity 94%, Specificity 88%; LUS: Sensitivity 64%, Specificity 84% | VMT superior to LUS; combined assessment proposed diagnostic flow chart; potentially useful for non-cardiologists |
| Y. Tian et al. | 2020 | Bioinformatic diagnostic model study | NA | HF (unspecified phenotype) | NA | Random forest + artificial neural network using gene expression data | Six key genes identified; model validated in public datasets | Novel genes (CSDC2, FREM1, ZMAT1) linked to HF; promising diagnostic model |
| Y. Tomonaga et al. | 2011 | Prospective multicentre controlled trial cluster-randomised | 369 | Suspected HF (among ACS/TE/HF presentations) | Primary Care | 3-in-1 POCT: cTnT, NT-proBNP, D-dimer vs conventional diagnosis | Working diagnoses more accurate in POCT group overall (75.7% vs 59.6%) and for ACS/HF/TE (69.8% vs 45.2%) | POCT improves diagnostic accuracy in primary care; significant benefit |
| Y. Wan et al. | 2015 | Diagnostic accuracy study | 98 | HF (unspecified phenotype) | Cardiology outpatient | Plasma NT-proBNP and proBNP assays (various antibody epitopes) alone or in combination | NT-proBNP13-76 and combined assays improved diagnostic performance over commercial tests; proBNP assay acceptable | Small sample; experimental assays; findings need validation in clinical trials |
| Y. Wang et al. | 2018 | Diagnostic study | 290 | Stage B HF (asymptomatic LV dysfunction) | General population (with T2DM) | Echocardiographic assessment (LVH, LAE, DD, GLS) to refine Stage B HF criteria | LVH and impaired GLS (<16%) independently predicted incident HF | GLS added diagnostic value beyond LVH and clinical risk; short follow-up (1.5 years); |
| Y. Zhang | 2023 | Retrospective case-control study | 145 | Chronic HF | Hospital/ outpatient cardiology | echocardiography (Echo) and serum C-reactive protein (CRP) levels | Combined ROC analysis shows high diagnostic value; | LVEF, FS, E/A and CRP are independent risk factors for CHF1 |
| Z. Huang et al. | 2020 | Systematic review and meta-analysis | 45 studies | HF (unspecified phenotype) | Not specified | Novel biomarkers: copeptin, galectin-3, hs-cTnT, MR-proANP, MR-proADM, ST2 | hs-cTnT highest accuracy (AUC 0.89); MR-proADM lowest (AUC 0.68) | MR-proADM poor diagnostic capacity; combining biomarkers may improve accuracy |
| Z. J. Han et al. | 2015 | Systematic review and meta-analysis | 1654 | HF with pleural effusion | Not specified | Blood and pleural fluid natriuretic peptides (BNP, NT-proBNP, MR-proANP) | PF NT-proBNP: sensitivity 0.94, specificity 0.91; Blood NT-proBNP: sensitivity 0.92, specificity 0.88 | Effective diagnostic tools; further studies needed for MR-proANP and BNP1 |
